# Lysophospholipids Are Associated With Outcomes in Hospitalized Patients With Mild Traumatic Brain Injury

**DOI:** 10.1089/neu.2023.0046

**Published:** 2023-12-29

**Authors:** Aaron M. Gusdon, Jude PJ Savarraj, John B. Redell, Atzhiry Paz, Sarah Hinds, Angela Burkett, Glenda Torres, Xuefang Ren, Neeraj Badjatia, Georgene W. Hergenroeder, Anthony N. Moore, H. Alex Choi, Pramod K. Dash

**Affiliations:** ^1^ Division of Neurocritical Care, Department of Neurosurgery, McGovern School of Medicine, University of Texas Health Science Center, Houston, Texas, USA.; ^2^ Department of Neurobiology and Anatomy, McGovern School of Medicine, University of Texas Health Science Center, Houston, Texas, USA.; ^3^ Department of Neurology, University of Maryland School of Medicine, Baltimore, Maryland, USA.

**Keywords:** biomarkers, lysophospholipids, mild traumatic brain injury, outcomes

## Abstract

Mild traumatic brain injury (mTBI) accounts for 70–90% of all TBI cases. Lipid metabolites have important roles in plasma membrane biogenesis, function, and cell signaling. As TBI can compromise plasma membrane integrity and alter brain cell function, we sought to identify circulating phospholipid alterations after mTBI, and determine if these changes were associated with clinical outcomes. Patients with mTBI (Glasgow Coma Score [GCS] ≥13 and loss of consciousness <30 min) were recruited. A total of 84 mTBI subjects were enrolled after admission to a level I trauma center, with the majority having evidence of traumatic intracranial hemorrhage on brain computed tomography (CT). Plasma samples were collected within 24 h of injury with 32 mTBI subjects returning at 3 months after injury for a second plasma sample to be collected. Thirty-five healthy volunteers were enrolled as controls and had a one-time blood draw. Lipid metabolomics was performed on plasma samples from each subject. Fold change of selected lipid metabolites was determined. Multivariable regression models were created to test associations between lipid metabolites and discharge and 6-month Glasgow Outcomes Scale-Extended (GOSE) outcomes (dichotomized between “good” [GOSE ≥7] and “bad” [GOSE ≤6] functional outcomes). Plasma levels of 31 lipid metabolites were significantly associated with discharge GOSE using univariate models; three of these metabolites were significantly increased, while 14 were significantly decreased in subjects with good outcomes compared with subjects with poor outcomes. In multivariable logistic regression models, higher circulating levels of the lysophospholipids (LPL) 1-linoleoyl-glycerophosphocholine (GPC) (18:2), 1-linoleoyl-GPE (18:2), and 1-linolenoyl-GPC (18:3) were associated with both good discharge GOSE (odds ratio [OR] 12.2 [95% CI 3.35, 58.3], *p* = 5.23 × 10^−4^; OR 9.43 [95% CI 2.87, 39.6], *p* = 7.26 × 10^−4^; and OR 5.26 [95% CI 1.99, 16.7], *p* = 2.04 × 10^−3^, respectively) and 6-month (OR 4.67 [95% CI 1.49, 17.7], *p* = 0.013; OR 2.93 [95% CI 1.11, 8.87], *p* = 0.039; and OR 2.57 [95% CI 1.08, 7.11], *p* = 0.046, respectively). Compared with healthy volunteers, circulating levels of these three LPLs were decreased early after injury and had normalized by 3 months after injury. Logistic regression models to predict functional outcomes were created by adding each of the described three LPLs to a baseline model that included age and sex. Including 1-linoleoyl-GPC (18:2) (8.20% improvement, *p* = 0.009), 1-linoleoyl-GPE (18:2) (8.85% improvement, *p* = 0.021), or 1-linolenoyl-GPC (18:3) (7.68% improvement, *p =* 0.012), significantly improved the area under the curve (AUC) for predicting discharge outcomes compared with the baseline model. Models including 1-linoleoyl-GPC (18:2) significantly improved AUC for predicting 6-month outcomes (9.35% improvement, *p* = 0.034). Models including principal components derived from 25 LPLs significantly improved AUC for prediction of 6-month outcomes (16.0% improvement, *p =* 0.020). Our results demonstrate that higher plasma levels of LPLs (1-linoleoyl-GPC, 1-linoleoyl-GPE, and 1-linolenoyl-GPC) after mTBI are associated with better functional outcomes at discharge and 6 months after injury. This class of phospholipids may represent a potential therapeutic target.

## Introduction

Traumatic brain injury (TBI) results in ∼2,500,000 hospitalizations, and accounts for one third of the injury-related deaths in the United States annually.^1^ TBI results in significant morbidity, with at least 3,200,000–5,300,000 persons living with TBI-related disability, including cognitive and neuropsychiatric sequelae.^2^ The incidence of mild TBI (mTBI) has been estimated at 200–300/100,000 persons per year. The actual incidence is likely to be significantly higher, as many individuals who have incurred a mTBI do not present to the hospital.^3^ mTBI presents as a clinical spectrum ranging from brief confusion with rapid recovery to loss of consciousness (LOC) for up to 30 min and post-traumatic amnesia.^4,5^ Although some mTBI patients may exhibit prolonged symptoms (e.g., dizziness, fatigue, headaches, short-term memory loss), mild symptoms and a lack of objective imaging findings can lead to a missed diagnosis in other patients.^6^ A more thorough understanding of the systemic pathophysiological changes that occur after a mTBI will allow for better detection of injury, prediction of outcomes, and identification of therapeutic targets.

Lipids are essential for neuronal membrane integrity and signaling. Primary injury from the mechanical forces of the trauma itself transiently stretches plasma membranes and axons, triggering cellular, molecular, and neurochemical changes that can set into motion secondary injury processes that worsen neuropsychological outcomes. Oxidative damage is an important secondary injury process that can lead to plasma membrane phospholipid hydrolysis and has been proposed to contribute to neuronal dysfunction and cognitive impairments.^7,8^ Neuronal repair after injury is likely to require synaptic remodeling and replenishment of damaged membrane phospholipids.^9^ Phosphatidylcholines (PC) are an important class of phospholipids that are synthesized via the Kennedy cycle and are required for membrane repair.^10^ There has been considerable interest in the use of the Kennedy cycle intermediate cytidine-5’-diphosphate choline (CDP-choline) for the treatment of TBI and other neurological disorders.^11–13^ It has been postulated that providing CDP-choline may be able to augment brain phospholipid metabolism and restore neuronal function after an insult.^14,15^

Free fatty acids (FFAs) are required for the synthesis of phospholipids for membrane repair,^16^ and are also associated with a shift toward glycolytic metabolism.^17,18^ However, increased levels of FFAs may result in lipotoxicity via mechanisms such as lipid peroxidation.^19,20^ Polyunsaturated fatty acids (PUFAs) are a subset of FFAs that are incorporated into membrane phospholipids, have important anti-inflammatory effects, and may play a role in recovery from TBI.^21^ Dietary PUFAs have been shown to improve neurological recovery after TBI.^22^ A few recent studies have reported changes in the composition of circulating lipids after a TBI, as well as associations with disease severity and clinical outcomes.^23–25^ For example, Thomas and coworkers recently reported that changes in the levels of 19 metabolites, including lipids measured within 24 h of TBI, were predictive of 6-month Glasgow Outcome Scale – Extended (GOSE) with area under the curve (AUC) ranging from 0.68 to 0.73.^24^ These metabolites included PCs, lysophospholipids (LPLs), and sphingomyelins (SMs).

Herein, we assessed lysophospholipid metabolites in plasma samples from mTBI patients early (within 24 h) and late (3 months) after injury, as well as healthy controls, to determine if altered levels were associated with outcome. We hypothesized that mTBI alters plasma lipid metabolites, and that these changes can be useful in predicting outcomes. Furthermore, identifying lipid metabolite profiles after mTBI may identify targets for therapeutic interventions that can improve outcomes.

## Methods

### Patient selection

mTBI patients were enrolled from a tertiary care center and level I trauma center, Memorial Hermann Hospital at the Texas Medical Center, Houston, TX, between December 2017 and August 2019. The study was approved by the institutional review board (IRB) at the University of Texas McGovern School of Medicine (HSC-MS-12-0637). All patients were evaluated by an attending neurointensivist and diagnosed with mTBI, defined as a Glasgow Coma Scale (GCS) score of ≥13 and LOC <30 minutes. Each patient received a computed tomography (CT) scan of the head as part of routine clinical evaluation. Of the 84 TBI patients, 6 had no acute CT findings (7.14%), while the rest had intracranial findings including traumatic subarachnoid hemorrhage (*n* = 50, 59.5%), subdural hemorrhage (*n* = 47, 56.0%), epidural hemorrhage (*n* = 3, 3.57%), or contusions (*n* = 20, 23.8%). Multi-compartment hemorrhage was common (*n* = 31, 36.9%). No patients had significant mass effect or midline shift, and none required surgical intervention. A description of the types of hemorrhages found in each patient is provided in Table S1. Healthy volunteers were enrolled from outpatient clinics at the University of Texas Health Science Center and the University of Maryland Medical Center, and had no neurological diagnoses.

### Demographic, clinical, and outcome data

Demographic data were collected for each subject including age, sex, and medical comorbidities. The modified Rankin Score (mRS) at discharge was assessed by the attending physician taking care of the patient.^26^ The GOSE is one of the most widely used metrics of disability and recovery after TBI.^27^ GOSE outcomes range from 1 to 8 (1 = dead, and 8 = upper good recovery); the full scale is shown in Table S2. GOSE was assessed at discharge by the attending physician caring for the patient. GOSE and mRS at 6 months post-injury were assessed via follow-up phone call by trained research personnel using standardized questionnaires.

### Sample collection and processing

Blood samples were collected in ethylenediaminetetraacetic acid (EDTA)-containing tubes and centrifuged at 1000*g* for 10 min at 4°C to remove circulating cells and isolate the plasma fraction. All mTBI subjects had a plasma sample collected within 24 h after injury. Thirty-two of these mTBI subjects returned 3 months after injury (3.16 ± 1.05 months) to provide a second sample. For the healthy volunteer subjects (*n* = 35), a single blood draw was obtained and processed at enrollment. Plasma samples were stored at -80°C until used.

### Metabolomics

Metabolomics was performed by Metabolon, Inc (Morrisville, NC) using ultra-high-performance liquid chromatography-tandem mass spectrometry (UPLC-MS/MS) as previously described.^28^ Metabolites were identified based on comparisons of each ion with a reference library.^29^ The AUC for each metabolite peak was quantified and used in the fold-change calculations. A total of 318 lipid metabolites were identified and analyzed (Table S3). Lipid metabolites included sphingolipids and ceramides; acyl-carnitines; intermediates of fatty acid metabolism (acyl-choline, acyl-glutamine, acyl-glycine); fatty acids (short, intermediate, and long chain fatty acids; dicarboxylate, dihydroxyl, and monohydroxyl fatty acids; long-chain polyunsaturated (n3 and n6) fatty acids; PC, phosphatidylethanolamine (PE), phosphatidylinositol, and phosphatidylserine; and lysoplasmalogen and plasmalogen. Nomenclature for lipids includes the backbone and acyl chains in parentheses. Chemical structures were drawn with PubChem Sketcher V2.4 (https://pubchem.ncbi.nlm.nih.gov//edit3/index.html).

### Bioinformatics analyses

For each metabolite, fold change between comparison groups (e.g., “good” vs. “bad” dichotomized GOSE; mTBI vs. control) and corresponding raw and false discovery rate (FDR) corrected *p* values were calculated in R using MetaboAnalyst 5.0 (https://www.metaboanalyst.ca).^30^ FDR corrected *p* values were considered to be significant at *p* < 0.05. Pearson correlation coefficients were calculated for metabolites of interest.

### Statistical analysis

Descriptive statistics (medians and interquartile ranges [IQR] or percentages) were calculated for each subject. Categorical variables were compared using Pearson χ^2^ test, while continuous variables were compared using a Mann–Whitney test. For descriptive statistics, *p* values were two-sided and considered statistically significant if *p* < 0.05. All statistical analyses were performed in R (R Foundation for Statistical Computing, version 4.1.2).

### Predictive models

Functional outcomes at discharge and 6 months after discharge were quantified using GOSE scores dichotomized as a binary response: “good” (GOSE ≥7) and “bad” (GOSE ≤6). Principal components analysis (PCA) was performed on metabolites comprising a particular pathway producing a new set of variables named “principal components,” and eliminating redundant dimensions, with original variables combined in each principal component.^31,32^ PCA prevents overfitting by reducing the number of dimensions within the predictors. The “PCAtools” package in R was used to generate scree plots with the “elbow” method used to select the number of PCs needed to account for at least 80% of the variation in the data set.^33,34^ We developed a baseline logistic regression model for mTBI patients (including age and sex) and predictive models (logistic regression models that included individual lipid metabolites or principal components in addition to baseline parameters). Models were developed to predict functional outcomes at discharge and 6 months after discharge. The area under the receiver operating curves (ROC) of the predictions was calculated, and the ROC curves were statistically compared using the DeLong test.^35^ Models were developed using the “scikitlearn” toolbox available in Python (v3.6). Statistical analyses comparing ROC curves was performed using R (R Foundation for Statistical Computing, version 4.1.2).

## Results

### Demographics

Demographics for all subjects are shown in Table 1. A total of 35 control and 84 mTBI subjects were enrolled. Of the 84 mTBI subjects, 32 had a second, paired plasma sample obtained 3 months after injury. There were no significant differences in age or sex between the control and mTBI groups. No differences were seen between the percentage of mTBI and control subjects with type 2 diabetes (T2DM) or hyperlipidemia (HLD). mTBI subjects had a median GCS of 15 (95% confidence interval [CI] 15, 15) with median GOSE at discharge and at 6 months after injury of 7 (95% CI 4,7) and 7 (95% CI 5,8), respectively. The median discharge mRS for the mTBI subjects was 1.0 (95% CI 1.0, 3.3). Among mTBI subjects, those with good compared with bad discharge GOSE were younger (median 46 years of age [IQR 29, 59] vs. 70 years of age [IQR 56, 76)], *p* = 3.19 × 10^−6^), but there was no difference in sex (Table 1). A significantly higher percentage of mTBI subjects with poor outcomes had self-reported HLD compared with mTBI subjects with good outcomes (*n* = 19 [51.4%] vs. *n* = *2* [25.5%], *p* = 0.022).

**Table 1.** Subject Demographics

	Controls	TBI	*p *value*^c^*	TBI – good outcome	TBI – bad outcome	*p *value*^d^*
*n*	35	84		47	37	
Late time point		32				
Age^b^	56 (50, 66)	56 (40.3, 71.0)	0.436	46 (29, 59)	70 (56, 76)	3.19x10^−5^
Sex^a^	16 (45.7%)	29 (34.5%)	0.301	16 (34.0%)	13 (35.1%)	0.999
HLD^a^	10 (28.6%)	31 (36.9%)	0.407	12 (25.5%)	19 (51.4%)	0.022
T2DM^a^	8 (22.9%)	33 (39.3%)	0.095	18 (38.3%)	15 (40.5%)	0.999
GCS (Total)^b^		15 (15, 15)		15 (15, 15)	15 (15, 15)	0.010
GCS (Eyes)^b^		4 (4, 4)		4 (4, 4)	4 (4, 4)	0.049
GCS (Verbal)^b^		5 (5, 5)		5 (5, 5)	5 (5, 5)	0.113
GCS (Motor)^b^		6 (6, 6)		6 (6, 6)	6 (6, 6)	0.999
GOSE (discharge)^b^		7 (4, 7)		7 (7, 7)	4 (4, 5)	2.20x10^−16^
GOSE (6-months) ^b^		7 (5, 8)		7 (6, 8)	6 (4, 7)	0.00126
mRS (discharge) ^b^		1 (1, 3)		1 (1, 1)	4 (3, 4)	1.74x10^−15^

Data are presented as ^a^*n* (%) or ^b^median (interquartile range).

^c^ *P* value for the comparison between control and all TBI subjects.

^d^ *P* value for the comparison between TBI subjects with good or bad discharge outcomes. HLD, hyperlipidemia; T2DM, type 2 diabetes mellitus; GCS, Glasgow Coma Scale; GOSE, Glasgow Outcome Scale – Extended; mRS, modified Rankin Scale.

### Lipid metabolites associated with outcomes

**FIG. 1.**
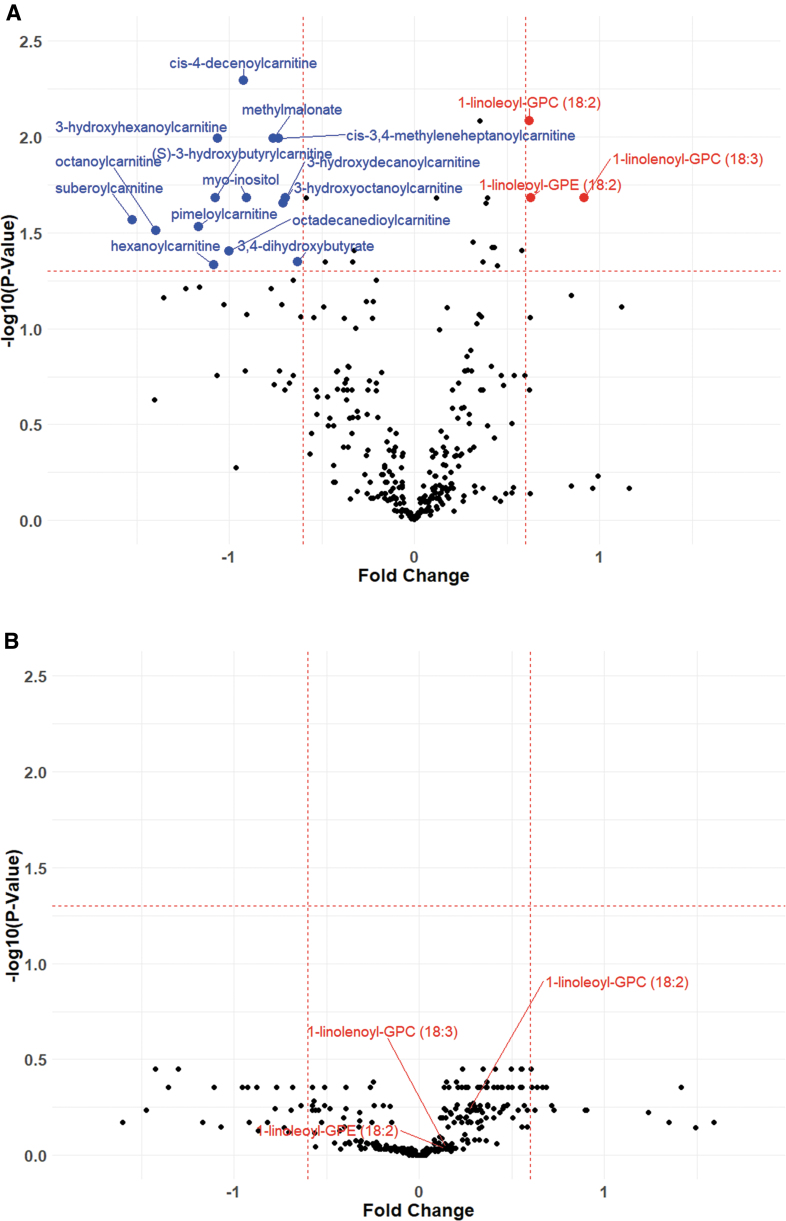
Circulating levels of lipid metabolites in relation to mild traumatic brain injury (mTBI) outcomes. mTBI patients were dichotomized by good or bad discharge Glasgow Outcome Scale Extended (GOSE), and plasma lipid metabolite fold changes were calculated for statistical comparison. Volcano plots demonstrating plasma lipid metabolite fold changes within 24 h **(A)** and 3 months **(B)** after mTBI. All *p* values are false discovery rate (FDR) corrected. Fold changes were considered to be significant at log_2_ (fold change) >0.6 or < -0.6, and corrected *p* < 0.05.

Functional outcomes were assessed by GOSE at discharge and at 6 months after discharge. We developed univariate models to assess associations between plasma lipid metabolites and dichotomized discharge and 6-month GOSE outcomes (“good” ≥ 7, “bad” ≤ 6) within the mTBI patient cohort. A total of 31 metabolites showed a significant association with discharge GOSE after FDR correction (Table S4). Of these metabolites, 3 were significantly increased (log_2_[FC] >0.6 and FDR-adjusted *p* value <0.05), and 14 were significantly decreased, in plasma samples obtained within 24 h of injury (log_2_[FC] < -0.6 and FDR-adjusted *p* value <0.05) (Fig. 1A; Table S4). mTBI subjects with good outcomes had higher levels of lysophosphatidylcholine (LPC) and lysophosphatidylethanolamine (LPE) and decreased levels of acyl-carnitines, as well as 3,4-dihydroxybutyrate. Discharge GOSE was not significantly associated with any metabolites at 3 months post-injury (Fig. 1B). Multivariable logistic regression models controlling for age, sex, race, ethnicity, and hyperlipidemia showed that out of the 17 metabolites differentially regulated within 24 h of mTBI, 3 (1-linoleoyl- glycerophosphorylcholine [GPC] [18:2], 1-linoleoyl-GPE [18:2], and 1-linolenoyl-GPC [18:3]) were independently associated with both discharge GOSE and 6-month GOSE (Table 2).

**Table 2.** Multivariable Models Assessing Associations Between Lysophospholipid Metabolites and Dichotomized GOSE Outcomes

		GOSE (discharge)*^a^*	GOSE (6-month)*^a^*
1-linoleoyl-GPC (18:2)	Unadjusted	8.75 (3.04, 30.3), ***p* = 1.87 × 10^−4^**	5.23 (1.87, 17.3), ***p* = 3.11 × 10^−3^**
	Adjusted	12.2 (3.35, 58.3), ***p* = 5.23 × 10^−4^**	4.67 (1.49, 17.7), ***p* = 0.013**
	Age	0.958 (0.921, 0.993), ***p* = 0.023**	0.952 (0.913, 0.988), ***p* = 0.013**
	Sex (male)	1.87 (0.549, 6.65), *p* = 0.318	0.206 (0.025, 1.19), *p* = 0.286
	Race (white)	1.42 (0.247, 8.27), *p* = 0.692	0.206 (0.025, 1.19), *p* = 0.098
	Ethnicity (Hispanic)	1.71 (0.499, 6.18), *p* = 0.397	1.23 (0.358, 4.34), *p* = 0.738
	HLD	0.580 (0.152, 2.14), *p* = 0.413	1.97 (0.524, 8.17), *p* = 0.327
1-linoleoyl-GPE (18:2)	Unadjusted	4.94 (2.06, 13.4), ***p* = 7.58 × 10^−4^**	2.40 (1.07, 5.84), ***p* = 0.041**
	Adjusted	9.43 (2.87, 39.6), ***p* = 7.26 × 10^−4^**	2.93 (1.11, 8.87), ***p* = 0.039**
	Age	0.954 (0.917, 0.988), ***p* = 0.012**	0.947 (0.909, 0.982), ***p* = 5.53 × 10^−3^**
	Sex (male)	2.44 (0.697, 9.23), *p* = 0.171	0.572 (0.152, 1.99), *p* = 0.388
	Race (white)	0.688 (0.107, 4.17), *p* = 0.683	0.153 (0.017, 0.949), *P* = 0.059
	Ethnicity (Hispanic)	1.80 (0.523, 6.50), *p* = 0.356	1.20 (0.358, 4.04), *p* = 0.770
	HLD	0.63 (0.168, 2.30), *p* = 0.481	2.16 (0.593, 8.83), *p* = 0.257
1-linolenoyl-GPC (18:3)	Unadjusted	4.60 (2.05, 12.2), ***p* = 7.11 × 10^−4^**	2.61 (1.26, 6.10), ***p* = 0.015**
	Adjusted	5.26 (1.99, 16.7), ***p* = 2.04 × 10^−3^**	2.57 (1.08, 7.11), ***p* = 0.046**
	Age	0.964 (0.929, 0.998), ***p* = 0.042**	0.955 (0.918, 0.989), ***p* = 0.016**
	Sex (male)	1.78 (0.542, 6.01), *p* 0.344	0.585 (0.158, 2.01), *p* = 0.403
	Race (white)	0.769 (0.121, 4.81), *p* = 0.776	0.162 (0.018, 1.08), *p* = 0.07
	Ethnicity (Hispanic)	1.78 (0.528, 6.31), *p* = 0.355	1.30 (0.383, 4.47), *p* = 0.674
	HLD	0.630 (0.176, 2.23), *p* = 0.471	1.96 (0.543, 7.85), *p* = 0.317

Bold values represent significant *p*-values < 0.05.

^a^ Data are presented as odds ratio (OR) with 95% confidence interval (CI). Levels of each lysophospholipid were log_10_ transformed.

HLD, hyperlipidemia; GOSE, Glasgow Outcome Scale – Extended

**FIG. 2.**
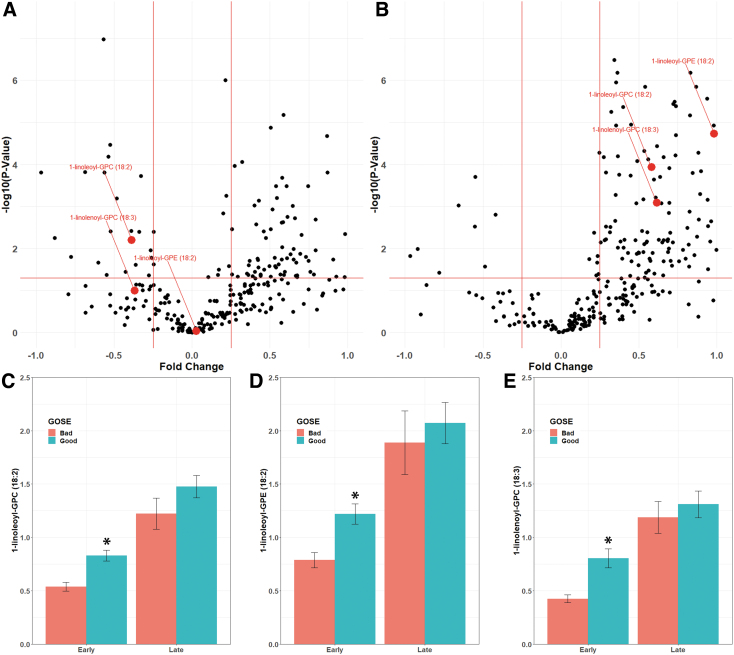
Changes in lipid metabolites over time after injury. Volcano plots of 24 h **(A)** and 3-month **(B)** changes after mild traumatic brain injury (mTBI). Fold changes are depicted comparing mTBI to controls. Metabolites found to be significantly higher in subjects with good outcomes (1-linoleoyl-GPC [18:2], 1-linoleoyl-GPE [18:2], and 1-linolenoyl-GPC [18:3]) are shown in red. Levels of each metabolite are shown in **C–E**. Levels are shown early (within 24 h) and late (3 months) after injury for those with bad (Glasgow Outcome Scale [GOSE] ≤6, red) and good (GOSE ≥7, blue) outcomes. *p < 0.001.

Lipid metabolites associated with outcomes showed significant changes between the early and late timepoints. Plasma levels of 1-linoleoyl-GPC (18:2) and 1-linolenoyl-GPC (18:3) were decreased acutely in mTBI patients relative to control subjects, while 1-linoleoyl-GPE (18:2) was not significantly changed (Fig. 2A). All three metabolites were significantly increased over control levels by 3 months after injury (Fig. 2B). mTBI patients with poor discharge GOSE had significantly lower levels of each of these three metabolites within 24 h of injury (1-linoleoyl-GPC [18:2]: *p =* 2.91 × 10^−5^; 1-linoleoyl-GPE [18:2]: *p* = 4.42 × 10^−4^; 1-linolenoyl-GPC [18:3]: *p* = 1.78 × 10^−4^; Fig. 2C–E) than did mTBI patients with good discharge GOSE. By 3 months post-injury, the plasma levels of these metabolites were elevated in mTBI subjects relative to controls. Similar to what was observed acutely after injury, plasma levels of these metabolites at 3 months had trends toward being lower in patients with poor GOSE, although these changes did not reach statistical significance. Levels of these three metabolites did not differ comparing mTBI subjects with or without HLD or T2DM (Table S5). No significant correlations were found between these three metabolites and body mass index (BMI) (Fig. S1).

### Correlations between plasma choline and phospholipid metabolites

**FIG. 3.**
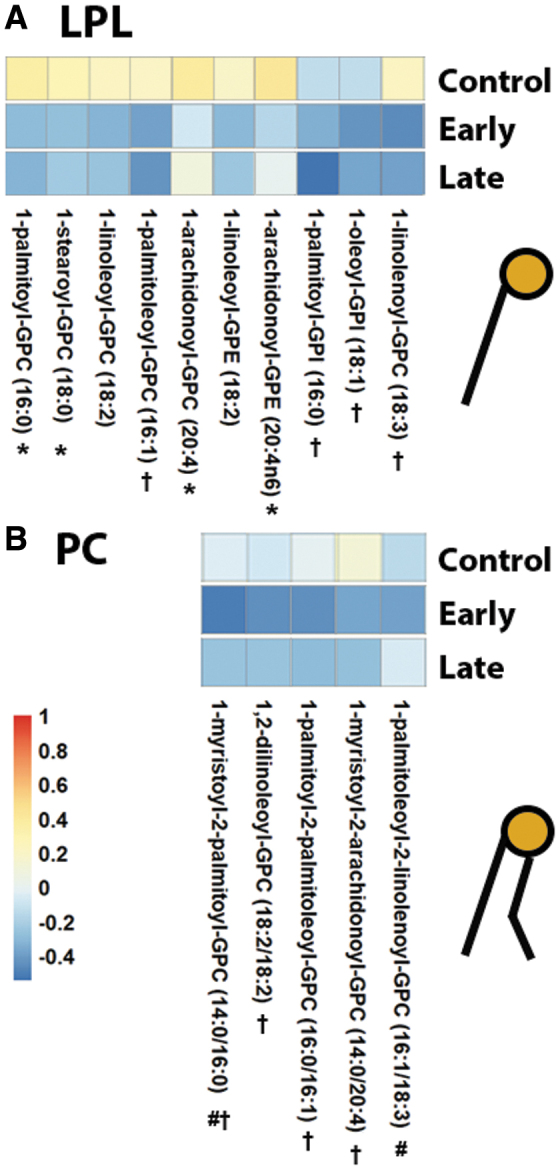
Associations between plasma choline and phospholipids. Each pixel represents a correlation coefficient corresponding to the phospholipid in that column and choline. Red, blue, and white coloring indicate positive, negative, or near-zero correlations. Correlations are shown between lysophosphlipids (LPL) and choline **(A)** and phosphatidylcholines (PC) and choline **(B)**. *p < 0.05 in controls, ^#^*p* < 0.05 early after mild traumatic brain injury (mTBI), ^†^*p* < 0.05 late after mTBI.

Choline is an important building block for the synthesis of phospholipids, including PC and PE. Plasma choline levels were not significantly different between control and 24 h mTBI plasma samples (log_2_[FC] = 0.15814, FDR *p* = 0.18). We next tested for correlations between LPLs metabolically downstream to choline as well as PCs (correlation coefficients and *p* values shown in Table S6). Choline was slightly positively correlated with most LPLs in control subjects, with significant positive correlations seen between choline and 1-palmitoyl-GPC (16:0) (ρ = 0.410, *p =* 0.010), 1-stearoyl-GPC (18:0) (ρ = 0.348, *p* = 0.030), 1-arachidonoyl-GPC (20:4) (ρ = 0.439, *p* = 0.005), and 1-arachidonoyl-GPE (20:4n6) (ρ = 462, *p* = 0.03) (Fig. 3A). These associations were more frequently negative after mTBI. Significant negative correlations were present between choline and 1-palmitoleoyl-GPC (16:1) (ρ = -0.433, *p* = 0.013), 1-palmitoyl-GPI (16:0) (ρ = -0.547, *p* = 0.001), 1-oleoyl-GPI (18:1) (ρ = -0.371, *p* = 0.036), and 1-linolenoyl-GPC (18:3) (ρ = -0.391, *p* = 0.027) late after mTBI (Fig. 3A). Similarly, choline was negatively correlated with PCs early (1-myristoyl-2-palmitoyl-GPC [14:0/16:0], ρ = -0.244, *p* = 0.025; and 1-linoleoyl-2-linolenoyl-GPC [18:2/18:3], ρ = -0.171, *p* = 0.023) and late (1-myrisotyl-2-palmitoyl-GPC [14:0/16:0], ρ = -0.371, *p* = 0.036; 1,2-dilinoleoyl-GPC [18:2/18:2], ρ = -0.374, *p* = 0.035; 1-palmitoyl-2-palmitoleoyl-GPC [16:0/16:1], ρ = -0.403, *p* = 0.022; and 1-myristoyl-2-arachidonoyl-GPC [14:0/20:4], ρ = -0.391, *p* = 0.027) after mTBI (Fig. 3B). It is of note that choline was positively correlated with GPC in controls (ρ = 0.355, *p* = 0.027) with trends toward negative correlations early and late after mTBI.

### Predictive models including circulating metabolites

Logistic regression models were developed using circulating metabolites to predict outcomes after mTBI. Outcomes were dichotomized as “good” (GOSE ≥7) or “bad” (GOSE ≤6). The baseline model included age and sex. Models were developed for both discharge and 6-month outcomes. Metabolites with significant differences between mTBI patients with good and bad outcomes in the 24 h post-injury plasma samples were added to baseline variables, and comparisons between ROC curves including only baseline variables, and baseline variables plus each metabolite, were made.

**FIG. 4.**
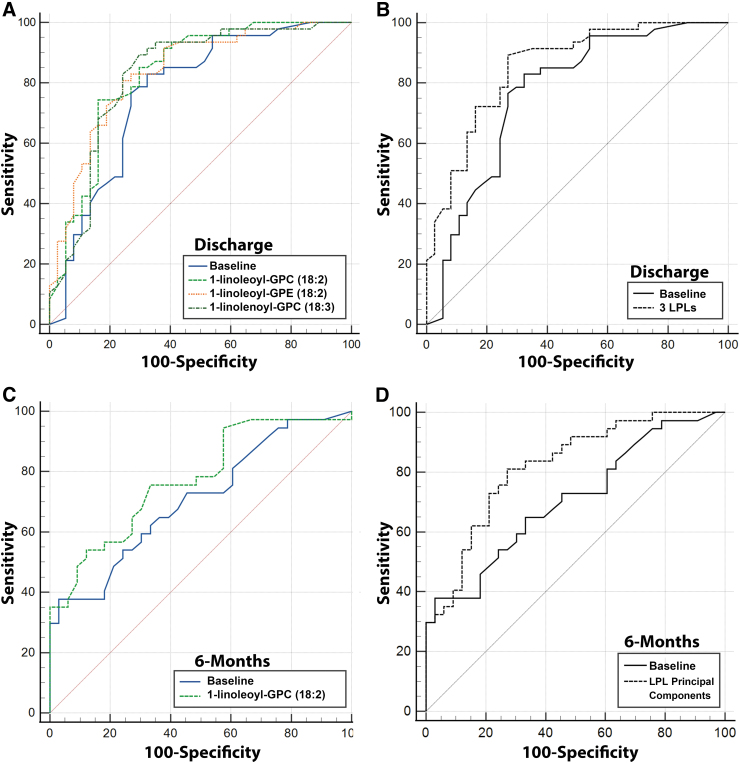
Receiver operating curve (ROC) analysis. Machine learning models were developed in order to determine the prognostic value of plasma lipid metabolites to predict outcomes after mild traumatic brain injury (mTBI). Baseline models included age and sex. Models were developed adding each of the three metabolites found to be upregulated in subjects with good discharge outcomes and to have significant associations with outcomes in multivariable models. Areas under the curve (AUCs) relative to the baseline model are shown for predicting discharge outcomes **(A)**. A model was also created including all three metabolites added to the baseline model to predict discharge outcomes **(B)**. Only one metabolite (1-linoleoyl-GPC [18:2]) when added to the baseline model individually significantly increased the AUC to predict 6-month outcomes **(C)**. Principal components (3) generated from 25 detected lysophosphlipid (LPL) metabolites were added to the baseline model to predict 6-month outcomes **(D)**.

The AUC for the baseline model to predict discharge GOSE was 0.768 (95% CI 0.663, 0.853) (Fig. 4A). Adding 1-linoleoyl-GPC (18:2) to the baseline model resulted in an 8.20% improvement in AUC to 0.831 (95% CI 0.734, 0.904), which was significantly higher than baseline (*p* = 0.0086). Adding 1-linoleoyl-GPE (18:2) to the baseline model resulted in an 8.85% improvement in AUC to 0.836 (95% CI 0.739, 0.908), which was significantly higher than baseline (*p* = 0.0206). Adding 1-linolenoyl-GPC (18:3) to the baseline model resulted in a 7.68% improvement in AUC to 0.827 (95% CI 0.729, 0.901), which was significantly higher than baseline (*p* = 0.0124). Adding all three LPL metabolites to the baseline model resulted in 11.33% improvement in AUC to 0.855 (95% CI 0.761, 0.922), which was significantly higher than baseline (*p* = 0.0108) (Fig. 4B).

The AUC for the baseline model to predict 6-month GOSE was 0.707 (95% CI 0.586, 0.810) (Fig. 4B). Adding 1-linoleoyl-GPC (18:2) to the baseline model resulted in a 9.34% improvement in AUC to 0.773 (95% CI 0.657, 0.865), which was significantly higher than baseline (*p* = 0.0339) (Fig. 4C). Adding 1-linoleoyl-GPE (18:2) to the baseline model resulted in an AUC of 0.745 (95% CI 0.627, 0.842), which was not significantly different than baseline (*p* = 0.176). Adding 1-linolenoyl-GPC (18:3) to the baseline model resulted in an AUC of 0.737 (0.618, 0.835), which was not significantly different than baseline (*p* = 0.1480). Adding all three LPL metabolites to the baseline model resulted in 9.49% improvement in AUC to 0.773 (95% CI 0.657, 0.864), which was not significantly different than baseline (*p* = 0.0809).

In order to assess the effect of the classes of metabolites, PCA was performed to reduce redundant dimensions, producing a new set of variables and preventing overfitting. PCA was performed using all 25 LPL metabolites detected and reduced into principal components. The “elbow” method was used to identify three PC variables that accounted for >80% of the variation in the data set.^33,34^ Adding these three LPL PC variables to the 6-month GOSE baseline model resulted in a 16.0% improvement in AUC to 0.820 (95% CI 0.710, 0.901), which was significantly higher than baseline (*p* = 0.0198) (Fig. 4D). PCA analysis was also performed on metabolites comprising PCs, phosphatidylethanolamines (PEs), polyunsaturated fatty acids (PUFAs), and diacylglycerols (DAGs). Addition of principal components from each of these pathways (or each metabolite individually) did not significantly increase the AUC over the baseline model for predicting either discharge or 6-month outcomes. ROC curves for each additional pathway assessed are shown in Figure S2 The metabolites comprising each pathway are shown in Table S7.

## Discussion

In the present study, we measured the levels of circulating lipids metabolites after mTBI. We show that changes in phospholipid metabolites occur early after injury, with acutely lower plasma levels of LPLs being associated with worse functional outcomes. Our findings suggest that changes in phospholipid metabolism after mTBI may provide insight into the pathophysiology of injury and identify future targets for therapeutic intervention.

**FIG. 5.**
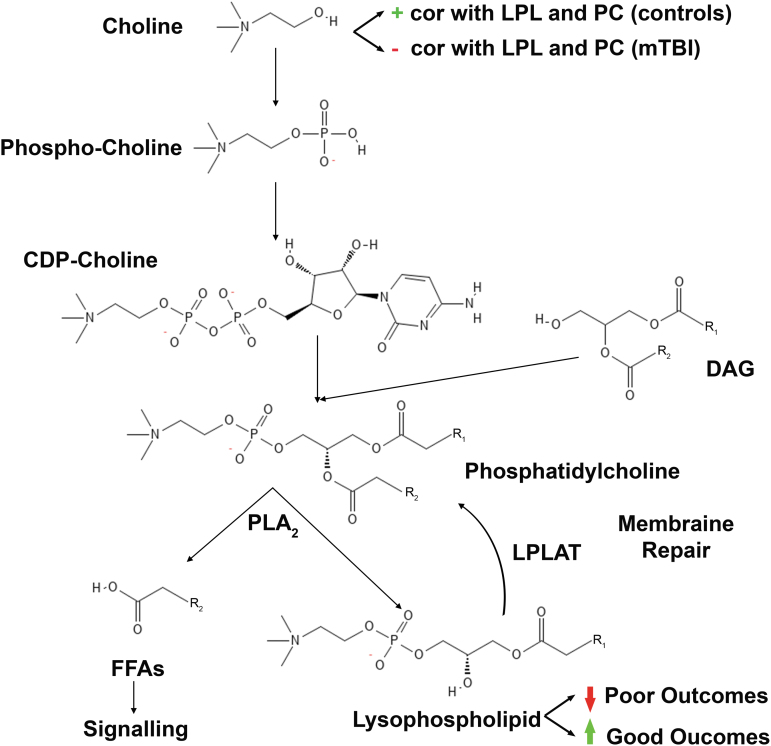
Schematic overview of pathways involved. A diagram of the Kennedy and Lands Cycles is shown depicting the biosynthesis pathways of membrane phosphatidylcholine. Phospholipases remove an acyl group from phosphatidylcholine to produce lysophospholipids and free fatty acids (FFAs). Lysophospholipids can be combined with diverse acyl groups to generate phosphatidylcholine for membrane repair. FFAs, such as polyunsaturated fatty acids (PUFAs), removed from the *sn-2* position of phosphatidylcholine may be used in downstream signaling pathways. An analogous pathway exists for phosphatidylethanolamine. Abbreviations: correlation (cor), lysophospholipid (LPL), phosphatidylcholine (PC), diacylglycerol (DAG), phospholipase A_2_ (PLA_2_), lysophospholipid acyltransferase (LPLAT).

Our main results demonstrate that decreased levels of the LPL class of phospholipids (namely, 1-linoleoyl-GPC [18:2], 1-linoleoyl-GPE [18:2], and linolenoyl-GPC [18:3]) early after mTBI are associated with poor outcomes. Interestingly, plasma levels of these LPLs were significantly elevated compared with control subjects by 3 months after injury. Figure 5 provides an overview of the key pathways required for membrane phospholipid synthesis and the structural similarity of the metabolites involved, as well as a summary of our main findings. Choline is a key precursor to phospholipid synthesis, and we showed that it was positively correlated with levels of LPLs in control subjects (Fig. 3A). These correlations were largely negative within 24 h of injury, suggesting the increased importance of circulating LPLs, which can regenerate PC and PE via the action of lysophospholipid acyltransferase (LPLAT) for membrane repair. It is therefore possible that bolstering early systemic synthesis of LPLs may be able to improve membrane repair and, subsequently, functional outcomes after injury.

Our findings are in line with results from previous studies demonstrating early decreases in circulating phospholipids, including those derived from choline, after TBI.^23,36,37^ Similarly, a decrease in phospholipids has been shown in a murine model of TBI, with lower levels associated with persistent sensorimotor and learning deficits.^38^ Recently, early decreases in phospholipids have been associated with poor outcomes after trauma, and administration of thawed plasma after injury resulted in a rebound of phospholipids levels.^37^ Importantly, supplementation with precursors of phospholipid synthesis after experimental TBI was shown to improve sensorimotor and cognitive outcomes,^16^ suggesting that the phospholipids may be involved in reparative mechanisms after TBI. PCs account for 40–50% of total phospholipids and naturally organize into lipid bilayers, accounting for the major constituent of plasma membranes.^39^ PEs are the second most common type of phospholipid and are enriched in the mitochondrial inner membrane.^40^ TBI causes damage to the white matter tracts, which require repair, creating an increased need for PC and PE synthesis. PC and PE are synthesized through the Kennedy cycle (Fig. 5).^10^ LPLs can also be used as substrates to generate PCs and PEs containing diverse acyl groups for membrane repair via the Lands cycle.^41^ We found negative correlations between choline and downstream PCs and LPLs in mTBI subjects, suggesting that membrane phospholipid synthesis from choline may be impaired after injury. Proinflammatory cytokines such as tumor necrosis factor (TNF)-α, which are over-expressed after TBI, can increase proteolysis of enzymes required for PC synthesis.^42,43^ The inability to synthesize PC in the presence of adequate substrates may explain the ineffectiveness of supplementation with upstream metabolites such as citicoline (CDP-choline) to improve outcomes after brain injury caused by stroke and TBI.^13,44^ This is consistent with our findings of similar levels of choline in controls compared with mTBI subjects. In order to increase PCs for membrane repair, a combination of LPLs, PUFAs, choline, vitamins, and other cofactors may be more effective.^16^

The lipid metabolites associated with functional outcomes (Table 2) predicted outcomes better than clinical variables alone in machine learning models (Fig. 4). These metabolites represent LPLs, which result from the removal of a fatty acid chain at the *sn-*2 position from PC or PE via the action of phospholipase A_2_ (PLA_2_).^45^ Membrane phospholipid composition is maintained through a series of de- and re-acylation reactions (Lands's cycle, as mentioned^41^). Saturated and monounsaturated fatty acyl chains are preferentially linked at the *sn*-1 position with PUFAs, preferentially at *sn*-2.^45^ It is believed that membrane phospholipids are metabolically active, resulting in constant remodeling and incorporation of PUFAs into PCs. The fatty acid makeup of membrane phospholipids affects membrane fluidity and curvature as well as signaling pathways that rely on membrane constituents.^46^ The reorganization of membrane lipids via Lands's cycle has recently been shown to play an important role following stroke, with the ratio of particular PCs to LPLs affecting neuronal function and markers of inflammation.^47^ LPLs also play a key role in transporting FFAs into the brain.^48^ Taken together, LPLs may play a pivotal role after injury in maintaining lipid membrane function, and possibly also for substrate transport into the central nervous system (CNS).

Our results are consistent with a recent report demonstrating an association between LPLs, including linoleoyl-GPC (18:2), and functional outcomes after mTBI.^24^ This study reported an AUC of 0.7008 for determining 6-month outcomes in this study. We also demonstrated a strong correlation between linoleoyl-GPC (18:2) and outcomes. Using a model containing principal components from 25 different LPLs resulted in an AUC of 0.820 for 6-month outcomes. Our results also show that although LPLs are decreased early after injury, levels are increased relative to controls by 3 months, suggesting a possible compensatory increase. In mouse models, dietary supplementation of LPLs increased brain ω-3 fatty acid content,^49^ and knockout of major facilitator superfamily domain containing 2a (Mfsd2a), which is necessary for LPL transport into the brain, results in decreased brain docosachexenoic acid (DHA) content, as well as cognitive impairment and neuronal death.^39,50^ LPLs administered enterally are able to be absorbed via the intestines to achieve bioavailability.^51^ Given the decreases in key LPLs seen early after injury in those subjects with poor outcomes, it is tempting to speculate that there may be a therapeutic role for early supplementation of LPLs or their components. However, future studies will be needed to address the efficacy of this approach in models of TBI.

All metabolites measured in this study were measured in plasma samples, raising the question of the tissue source of altered phospholipid metabolites. Studies have shown that TBI results in the release of lipid metabolites from injured membranes within the CNS.^8^ Phospholipases are activated within minutes after TBI, resulting in breakdown of membrane phospholipids leading to an accumulation of FFAs and DAGs. This calcium-dependent activation of phospholipases results in the generation of important lipid second messengers, but also likely has an effect on membrane function.^52^ The breakdown products of CNS membrane lipids may gain access to circulating blood, which could contribute to the changes in plasma lipids identified in our study. We suspect that lipid metabolites from peripheral tissues are key contributors to changes in plasma lipid levels after mTBI. An increased catabolic state occurs after trauma and other etiologies of critical illness.^53,54^ It is therefore possible that increased uptake and catabolism of lipids may account for the early decrease in lipids. Additionally, TBI affects fatty acid metabolism in liver and adipose tissue.^55^ Hepatic lipid droplets have been shown to accumulate in a rat model of TBI,^56^ via either increased activity of fatty acid synthase^57^ or increased hepatic uptake of FFAs via CD36.^58^ The stress response to trauma also results in the release of catecholamines and other hormones that can promote adipocyte triacylglycerol breakdown via lipolysis into glycerol and FFAs.^59,60^ FFAs such as DHA are pivotal for brain function, and are able to be taken up into the CNS from plasma pools.^61^ Future research will need to be conducted to determine the specific contributions of CNS vs peripheral lipids on the plasma differences found in this study.

The strengths of our study include our focused analysis of critical classes of lipid metabolites, assessment of lipids early and late after injury, and use of a population of mTBI subjects with validated clinical outcomes. However, this study has several important limitations. First, because all mTBI subjects are from one tertiary care center, generalizability may be limited. Further, whether these results can be generalized to other injury magnitudes, including concussion with normal CT findings, remains to be addressed. However, our results are consistent with those from other recent studies looking at mTBI. Second, all results were obtained from plasma samples and therefore it is not possible to determine the tissue and cellular origin of these metabolites. Third, this study consisted of a relatively small number of subjects with only a subset having samples available at the 3-month time point. Further studies in larger populations. as well as basic science studies in animal models to elucidate the mechanism of actions and contribution of the phospholipids identified in this study to mild TBI outcomes, will be needed to confirm these findings.

## Conclusions

In this study, we demonstrate that key lysophospholipid metabolites were decreased early after mTBI and were predictive of functional outcomes. These metabolites may be potential targets for therapeutic intervention.

## Transparency, Rigor, and Reproducibility Summary

This study was approved by the IRB at the University of Texas Health Science Center School of Medicine (HSC-MS-12-0637). Although our analysis plan was not formally pre-registered, the team members with primary responsibility for the analysis (A.M.G. and J.P.J.S.) certify that the analysis plan was pre-specified. A sample size of 84 TBI subjects was planned based on the availability of subjects with plasma and data available in our biorepository. The actual sample size was 84 subjects in the TBI group (with 32 subjects having a late time point available) with the inclusion of 35 control subjects based on availability and age and sex matching. All patients with TBI admitted to Memorial Hermann Hospital were approached for enrollment into our biobank. Among 311 TBI subjects with biosamples available, 84 subjects had mTBI as well as outcomes data, and were included in this study. Handling of biosamples and analysis was performed by team members blinded to relevant characteristics of the participants, and biomarkers were labeled using de-identified codes with a linking list required to obtained participant identifying information. Samples were acquired between December 2017 and August 2019. Samples were collected within 24 h from TBI and also at 3 months after TBI. Samples were collected at Memorial Hermann Hospital. Blood samples were collected in EDTA-containing tubes and were centrifuged to generate plasma. Samples were stored at -80°C prior to analysis. Aliquots were prepared prior to freezing to minimize freeze–thaw cycles. All mTBI samples were analyzed within the same batch. All analysis was performed by Metabolon, Inc (Morrisville, NC), and all equipment and reagents used were owned by Metabolon. The key inclusion criteria for mild TBI as well as outcome metrics (GOSE) are established standards in the field. Statistical tests used were based on the assumptions of non-normal distribution of the data. Multiple comparisons were addressed using an FDR correction, with significance after FDR correction set conservatively at 0.05. External validation studies are ongoing. De-identified data from this study are not currently available in a public archive. De-identified data from this study will be made available (as allowed by our IRB) to qualified investigators by e-mailing aaron.m.gusdon@uth.tmc.edu or p.dash@uth.tmc.edu. Analytical code used to conduct the analyses presented in this study are not available in a public repository. They are available by emailing aaron.m.gusdon@uth.tmc.edu or jude.p.savarraj@uth.tmc.edu. Future use of these biosamples is not allowed under our current IRB. The authors agree to publish the manuscript using the Mary Ann Liebert Inc. “Open Access” option with the appropriate license.

## Supplementary Material

Supplemental data

Supplemental data

Supplemental data

Supplemental data

Supplemental data

Supplemental data

Supplemental data

Supplemental data

Supplemental data
