# Supplementary material for: Lysophospholipids Are Associated With Outcomes in Hospitalized Patients With Mild Traumatic Brain Injury
Source: J Neurotrauma. 2023 Dec 29;41(1-2):59–72. doi: 10.1089/neu.2023.0046 (PMC11071087; doi:10.1089/neu.2023.0046)
Supplement: Supplemental data [file Suppl_TableS1.docx]

Supplementary Table S1: Computed tomography (CT) brain findings.

| **TBI Subject #** | **Negative** | **SAH** | **SDH** | **EDH** | **Contusion** | **Multi-compartment**  **hemorrhage** |
| --- | --- | --- | --- | --- | --- | --- |
| 1 | 1 | 0 | 0 | 0 | 0 | 0 |
| 2 | 0 | 1 | 0 | 0 | 0 | 0 |
| 3 | 0 | 1 | 1 | 0 | 0 | 1 |
| 4 | 0 | 0 | 1 | 0 | 0 | 0 |
| 5 | 1 | 0 | 0 | 0 | 0 | 0 |
| 6 | 0 | 1 | 1 | 0 | 0 | 1 |
| 7 | 0 | 0 | 1 | 0 | 0 | 0 |
| 8 | 0 | 1 | 0 | 0 | 0 | 0 |
| 9 | 0 | 1 | 1 | 0 | 0 | 1 |
| 10 | 0 | 1 | 1 | 0 | 0 | 1 |
| 11 | 0 | 1 | 0 | 0 | 0 | 0 |
| 12 | 0 | 0 | 0 | 0 | 1 | 0 |
| 13 | 0 | 1 | 0 | 0 | 0 | 0 |
| 14 | 0 | 1 | 0 | 1 | 0 | 1 |
| 15 | 0 | 0 | 1 | 0 | 0 | 0 |
| 16 | 0 | 1 | 1 | 0 | 1 | 1 |
| 17 | 0 | 0 | 1 | 0 | 1 | 1 |
| 18 | 0 | 1 | 1 | 0 | 1 | 1 |
| 19 | 1 | 0 | 0 | 0 | 0 | 0 |
| 20 | 0 | 0 | 1 | 0 | 0 | 0 |
| 21 | 0 | 1 | 0 | 0 | 0 | 0 |
| 22 | 0 | 1 | 0 | 0 | 0 | 0 |
| 23 | 0 | 1 | 1 | 0 | 1 | 1 |
| 24 | 0 | 1 | 0 | 0 | 0 | 0 |
| 25 | 0 | 0 | 1 | 0 | 0 | 0 |
| 26 | 0 | 1 | 0 | 0 | 0 | 0 |
| 27 | 0 | 1 | 0 | 1 | 0 | 1 |
| 28 | 1 | 0 | 0 | 0 | 0 | 0 |
| 29 | 0 | 0 | 1 | 0 | 0 | 0 |
| 30 | 0 | 1 | 1 | 0 | 1 | 1 |
| 31 | 0 | 1 | 0 | 0 | 0 | 0 |
| 32 | 0 | 1 | 0 | 0 | 0 | 0 |
| 33 | 0 | 1 | 1 | 0 | 0 | 1 |
| 34 | 0 | 0 | 1 | 0 | 1 | 1 |
| 35 | 0 | 1 | 0 | 1 | 0 | 1 |
| 36 | 0 | 0 | 1 | 0 | 0 | 0 |
| 37 | 0 | 0 | 1 | 0 | 0 | 0 |
| 38 | 0 | 1 | 1 | 0 | 1 | 1 |
| 39 | 0 | 1 | 0 | 0 | 0 | 0 |
| 40 | 0 | 1 | 0 | 0 | 0 | 0 |
| 41 | 0 | 1 | 1 | 0 | 0 | 1 |
| 42 | 1 | 0 | 0 | 0 | 0 | 0 |
| 43 | 0 | 1 | 0 | 0 | 0 | 0 |
| 44 | 0 | 0 | 1 | 0 | 0 | 0 |
| 45 | 0 | 1 | 0 | 0 | 0 | 0 |
| 46 | 1 | 0 | 0 | 0 | 0 | 0 |
| 47 | 0 | 1 | 0 | 0 | 0 | 0 |
| 48 | 0 | 1 | 1 | 0 | 0 | 1 |
| 49 | 0 | 1 | 0 | 0 | 1 | 1 |
| 50 | 0 | 0 | 1 | 0 | 1 | 1 |
| 51 | 0 | 0 | 1 | 0 | 0 | 0 |
| 52 | 0 | 0 | 1 | 0 | 0 | 0 |
| 53 | 0 | 0 | 1 | 0 | 0 | 0 |
| 54 | 0 | 1 | 0 | 0 | 0 | 0 |
| 55 | 0 | 1 | 0 | 0 | 0 | 0 |
| 56 | 0 | 1 | 0 | 0 | 1 | 1 |
| 57 | 0 | 0 | 1 | 0 | 0 | 0 |
| 58 | 0 | 1 | 1 | 0 | 1 | 1 |
| 59 | 0 | 1 | 0 | 0 | 0 | 0 |
| 60 | 0 | 1 | 0 | 0 | 0 | 0 |
| 61 | 0 | 1 | 1 | 0 | 1 | 1 |
| 62 | 0 | 1 | 1 | 0 | 1 | 1 |
| 63 | 0 | 1 | 1 | 0 | 0 | 1 |
| 64 | 0 | 0 | 0 | 0 | 1 | 0 |
| 65 | 0 | 1 | 1 | 0 | 0 | 1 |
| 66 | 0 | 0 | 1 | 0 | 0 | 0 |
| 67 | 0 | 1 | 0 | 0 | 0 | 0 |
| 68 | 0 | 0 | 1 | 0 | 0 | 0 |
| 69 | 0 | 0 | 1 | 0 | 0 | 0 |
| 70 | 0 | 1 | 1 | 0 | 0 | 1 |
| 71 | 0 | 0 | 1 | 0 | 0 | 0 |
| 72 | 0 | 0 | 0 | 0 | 1 | 0 |
| 73 | 0 | 0 | 1 | 0 | 0 | 0 |
| 74 | 0 | 1 | 1 | 0 | 1 | 1 |
| 75 | 0 | 1 | 0 | 0 | 1 | 1 |
| 76 | 0 | 1 | 1 | 0 | 1 | 1 |
| 77 | 0 | 1 | 0 | 0 | 0 | 0 |
| 78 | 0 | 1 | 1 | 0 | 0 | 1 |
| 79 | 0 | 0 | 1 | 0 | 0 | 0 |
| 80 | 0 | 0 | 1 | 0 | 0 | 0 |
| 81 | 0 | 1 | 0 | 0 | 0 | 0 |
| 82 | 0 | 1 | 1 | 0 | 1 | 1 |
| 83 | 0 | 0 | 1 | 0 | 0 | 0 |
| 84 | 0 | 0 | 1 | 0 | 0 | 0 |
| **Total:** | **6 (7.14%)** | **50 (59.5)** | **47 (56.0%)** | **3 (3.57%)** | **20 (23.8%)** | **31 (36.9%)** |

0 indicates absence of lesion, while 1 indicates presence of lesion. Abbreviations: SAH (subarachnoid hemorrhage), SDH (subdural hematoma), EDH (epidural hematoma).
