# Supplementary material for: Lysophospholipids Are Associated With Outcomes in Hospitalized Patients With Mild Traumatic Brain Injury
Source: J Neurotrauma. 2023 Dec 29;41(1-2):59–72. doi: 10.1089/neu.2023.0046 (PMC11071087; doi:10.1089/neu.2023.0046)
Supplement: Supplemental data [file Suppl_TableS2.docx]

Supplementary Table S2: Glasgow Outcomes Scale-Extended (GOSE).

| **GOSE Score** | **Outcome** | **Description** |
| --- | --- | --- |
| 1 | Dead | Dead |
| 2 | Vegetative State | Unconscious, no awareness with only reflex responses with periods of spontaneous eye opening |
| 3 | Lower Severe Disability | Dependent, requiring help with all activities of daily life, required constant assistance and not able to be left alone |
| 4 | Upper Severe Disability | Dependent, but able to be left at home for up to eight hours |
| 5 | Lower Moderate Severity | Able to return to work in a sheltered or non-competitive capacity, daily psychological symptoms |
| 6 | Upper Moderate Severity | Returned to work at reduced capacity, with frequent psychological symptoms |
| 7 | Lower Good Recovery | Returned to normal life and work with some psychological symptoms |
| 8 | Upper Good Recovery | Fully recovered and returned to normal life |
