# Supplementary material for: Lysophospholipids Are Associated With Outcomes in Hospitalized Patients With Mild Traumatic Brain Injury
Source: J Neurotrauma. 2023 Dec 29;41(1-2):59–72. doi: 10.1089/neu.2023.0046 (PMC11071087; doi:10.1089/neu.2023.0046)
Supplement: Supplemental data [file Suppl_TableS3.docx]

Supplementary Table S3: Metabolites assayed

|  | CHEMICAL NAME | PATHWAY | HMDB | KEGG | PUBCHEM |
| --- | --- | --- | --- | --- | --- |
| 1 | 9,10-DiHOME | Fatty Acid, Dihydroxy | HMDB0004704 | C14828 | 9966640 |
| 2 | linoleate (18:2n6) | Long Chain Polyunsaturated Fatty Acid (n3 and n6) | HMDB0006270, HMDB0000673 | C01595 | 5280450 |
| 3 | laurate (12:0) | Medium Chain Fatty Acid | HMDB0000638 | C02679 | 3893 |
| 4 | arachidonate (20:4n6) | Long Chain Polyunsaturated Fatty Acid (n3 and n6) | HMDB0001043 | C00219 | 444899 |
| 5 | choline phosphate | Phospholipid Metabolism | HMDB0001565 | C00588 | 1014 |
| 6 | sphingosine | Sphingosines | HMDB0000252 | C00319 | 5280335 |
| 7 | sphinganine | Sphingolipid Synthesis | HMDB0000269 | C00836 | 3126 |
| 8 | behenate (22:0)* | Long Chain Saturated Fatty Acid | HMDB0000944 | C08281 | 8215 |
| 9 | glutarate (C5-DC) | Fatty Acid, Dicarboxylate | HMDB0000661 | C00489 | 7434418048 |
| 10 | myo-inositol | Inositol Metabolism | HMDB0000211 | C00137 | 892 |
| 11 | methylmalonate (MMA) | Fatty Acid Metabolism (also BCAA Metabolism) | HMDB0000202 | C02170 | 487 |
| 12 | palmitate (16:0) | Long Chain Saturated Fatty Acid | HMDB0000220 | C00249 | 985 |
| 13 | pelargonate (9:0) | Medium Chain Fatty Acid | HMDB0000847 | C01601 | 8158 |
| 14 | stearate (18:0) | Long Chain Saturated Fatty Acid | HMDB0000827 | C01530 | 5281 |
| 15 | palmitoleate (16:1n7) | Long Chain Monounsaturated Fatty Acid | HMDB0003229 | C08362 | 445638 |
| 16 | myristate (14:0) | Long Chain Saturated Fatty Acid | HMDB0000806 | C06424 | 11005 |
| 17 | malonate | Fatty Acid Synthesis | HMDB0000691 | C00383 | 867 |
| 18 | caprate (10:0) | Medium Chain Fatty Acid | HMDB0000511 | C01571 | 2969 |
| 19 | margarate (17:0) | Long Chain Saturated Fatty Acid | HMDB0002259 | NA | 10465 |
| 20 | nonadecanoate (19:0) | Long Chain Saturated Fatty Acid | HMDB0000772 | C16535 | 12591 |
| 21 | arachidate (20:0) | Long Chain Saturated Fatty Acid | HMDB0002212 | C06425 | 10467 |
| 22 | N-stearoyl-sphinganine (d18:0/18:0)* | Dihydroceramides | HMDB0011761 | NA | 5283573 |
| 23 | heptanoate (7:0) | Medium Chain Fatty Acid | HMDB0000666 | C17714 | 8094 |
| 24 | caproate (6:0) | Medium Chain Fatty Acid | HMDB0000535 | C01585 | 8892 |
| 25 | caprylate (8:0) | Medium Chain Fatty Acid | HMDB0000482 | C06423 | 379 |
| 26 | pentadecanoate (15:0) | Long Chain Saturated Fatty Acid | HMDB0000826 | C16537 | 13849 |
| 27 | phosphoethanolamine | Phospholipid Metabolism | HMDB0000224 | C00346 | 1015 |
| 28 | erucate (22:1n9) | Long Chain Monounsaturated Fatty Acid | HMDB0002068 | C08316 | 8.2165E+10 |
| 29 | butyrate/isobutyrate (4:0) | Short Chain Fatty Acid | HMDB0000039 | C00246 | 264 |
| 30 | dihomo-linoleate (20:2n6) | Long Chain Polyunsaturated Fatty Acid (n3 and n6) | HMDB0005060 | C16525 | 6439848 |
| 31 | 2-hydroxystearate | Fatty Acid, Monohydroxy | HMDB0062549 | C03042, C03045 | 4.3989E+16 |
| 32 | glycerol | Glycerolipid Metabolism | HMDB0000131 | C00116 | 753 |
| 33 | choline | Phospholipid Metabolism | HMDB0000097 | C00114 | 305 |
| 34 | N-palmitoyl-sphingosine (d18:1/16:0) | Ceramides | HMDB0004949 | NA | 5283564 |
| 35 | 1-palmitoyl-2-oleoyl-GPE (16:0/18:1) | Phosphatidylethanolamine (PE) | HMDB0005320 | NA | 5283496 |
| 36 | 1-palmitoyl-2-linoleoyl-GPI (16:0/18:2) | Phosphatidylinositol (PI) | HMDB0009784 | NA | 46891795 |
| 37 | 1-palmitoyl-2-linoleoyl-GPC (16:0/18:2) | Phosphatidylcholine (PC) | HMDB0007973 | NA | 5287971 |
| 38 | stearoyl sphingomyelin (d18:1/18:0) | Sphingomyelins | HMDB0001348 | C00550 | 6453725 |
| 39 | 1-palmitoyl-2-oleoyl-GPC (16:0/18:1) | Phosphatidylcholine (PC) | HMDB0007972 | C13875 | 6436017 |
| 40 | N-stearoyl-sphingosine (d18:1/18:0)* | Ceramides | HMDB0004950 | NA | 5283565 |
| 41 | azelate (C9-DC) | Fatty Acid, Dicarboxylate | HMDB0000784 | C08261 | 2266 |
| 42 | eicosapentaenoate (EPA; 20:5n3) | Long Chain Polyunsaturated Fatty Acid (n3 and n6) | HMDB0001999 | C06428 | 446284 |
| 43 | carnitine | Carnitine Metabolism | HMDB0000062 | C00487,C00308 | 288 |
| 44 | glycerol 3-phosphate | Glycerolipid Metabolism | HMDB0000126 | C0093 | 754 |
| 45 | glycerophosphorylcholine (GPC) | Phospholipid Metabolism | HMDB0000086 | C00670 | 71920 |
| 46 | 1-stearoyl-2-arachidonoyl-GPI (18:0/20:4) | Phosphatidylinositol (PI) | HMDB0009815 | NA | NA |
| 47 | sphingosine 1-phosphate | Sphingosines | HMDB0000277 | C06124 | 5283560 |
| 48 | 1-stearoyl-2-oleoyl-GPS (18:0/18:1) | Phosphatidylserine (PS) | HMDB0010163 | NA | 9547087 |
| 49 | 1-stearoyl-GPI (18:0) | Lysophospholipid | HMDB0240261 | NA | 7.1581E+15 |
| 50 | 1,2-dipalmitoyl-GPC (16:0/16:0) | Phosphatidylcholine (PC) | HMDB0000564 | D03585 | 452110 |
| 51 | docosahexaenoate (DHA; 22:6n3) | Long Chain Polyunsaturated Fatty Acid (n3 and n6) | HMDB0002183 | C06429 | 445580 |
| 52 | 1-myristoyl-2-palmitoyl-GPC (14:0/16:0) | Phosphatidylcholine (PC) | HMDB0007869 | NA | 129657 |
| 53 | maleate | Fatty Acid, Dicarboxylate | HMDB0000176 | C01384 | 444266 |
| 54 | 2-hydroxyoctanoate | Fatty Acid, Monohydroxy | HMDB0002264 | NA | 9.4181E+18 |
| 55 | 3-hydroxyoctanoate | Fatty Acid, Monohydroxy | HMDB0001954 | C20793 | 26613 |
| 56 | palmitoylcarnitine (C16) | Fatty Acid Metabolism (Acyl Carnitine, Long Chain Saturated) | HMDB0000222 | C02990 | 461 |
| 57 | hexanoylcarnitine (C6) | Fatty Acid Metabolism (Acyl Carnitine, Medium Chain) | HMDB0000756 | NA | 6426853 |
| 58 | acetylcarnitine (C2) | Fatty Acid Metabolism (Acyl Carnitine, Short Chain) | HMDB0000201 | C02571 | 7045767 |
| 59 | 1-palmitoylglycerol (16:0) | Monoacylglycerol | HMDB0011564 | NA | 14900 |
| 60 | 1-oleoylglycerol (18:1) | Monoacylglycerol | HMDB0011567 | NA | 5283468 |
| 61 | 2-oleoylglycerol (18:1) | Monoacylglycerol | HMDB0011537 | NA | 5319879 |
| 62 | 2-linoleoylglycerol (18:2) | Monoacylglycerol | HMDB0011538 | NA | 5365676 |
| 63 | 3-hydroxydecanoate | Fatty Acid, Monohydroxy | HMDB0002203 | NA | 26612 |
| 64 | 1-linoleoylglycerol (18:2) | Monoacylglycerol | HMDB0011568 | NA | 5283469 |
| 65 | butyrylcarnitine (C4) | Fatty Acid Metabolism (also BCAA Metabolism) | HMDB0002013 | C02862 | 439829 |
| 66 | dodecanedioate (C12-DC) | Fatty Acid, Dicarboxylate | HMDB0000623 | C02678 | 12736 |
| 67 | 3-hydroxylaurate | Fatty Acid, Monohydroxy | HMDB0000387 | NA | 94216 |
| 68 | 3-hydroxysebacate | Fatty Acid, Monohydroxy | HMDB0000350 | NA | 3017884 |
| 69 | 5-hydroxyhexanoate | Fatty Acid, Monohydroxy | HMDB0000409, HMDB0000525 | NA | 170748 |
| 70 | propionylglycine | Fatty Acid Metabolism (also BCAA Metabolism) | HMDB0000783 | NA | 98681 |
| 71 | butyrylglycine | Fatty Acid Metabolism (also BCAA Metabolism) | HMDB0000808 | NA | 88412 |
| 72 | propionylcarnitine (C3) | Fatty Acid Metabolism (also BCAA Metabolism) | HMDB0000824 | C03017 | 107738 |
| 73 | 3-carboxy-4-methyl-5-propyl-2-furanpropanoate (CMPF) | Fatty Acid, Dicarboxylate | HMDB0061112 | NA | 123979 |
| 74 | docosapentaenoate (n3 DPA; 22:5n3) | Long Chain Polyunsaturated Fatty Acid (n3 and n6) | HMDB0006528, HMDB0001976 | C16513 | 6441454 |
| 75 | docosadienoate (22:2n6) | Long Chain Polyunsaturated Fatty Acid (n3 and n6) | HMDB0061714 | C16533 | 5282807 |
| 76 | adrenate (22:4n6) | Long Chain Polyunsaturated Fatty Acid (n3 and n6) | HMDB0002226 | C16527 | 5.2828E+13 |
| 77 | 10-undecenoate (11:1n1) | Medium Chain Fatty Acid | HMDB0033724 | C13910 | 14891 |
| 78 | myristoleate (14:1n5) | Long Chain Monounsaturated Fatty Acid | HMDB0002000 | C08322 | 5281119 |
| 79 | sebacate (C10-DC) | Fatty Acid, Dicarboxylate | HMDB0000792 | C08277 | 5192 |
| 80 | stearidonate (18:4n3) | Long Chain Polyunsaturated Fatty Acid (n3 and n6) | HMDB0006547 | C16300 | 5312508 |
| 81 | octanoylcarnitine (C8) | Fatty Acid Metabolism (Acyl Carnitine, Medium Chain) | HMDB0000791 | C02838 | 1.237E+13 |
| 82 | decanoylcarnitine (C10) | Fatty Acid Metabolism (Acyl Carnitine, Medium Chain) | HMDB0000651 | NA | 10245190 |
| 83 | 1-palmitoyl-GPC (16:0) | Lysophospholipid | HMDB0010382 | C04102 | 86554 |
| 84 | myristoylcarnitine (C14) | Fatty Acid Metabolism (Acyl Carnitine, Long Chain Saturated) | HMDB0005066 | NA | 6426854 |
| 85 | 1-stearoyl-GPC (18:0) | Lysophospholipid | HMDB0010384 | NA | 497300 |
| 86 | 1-oleoyl-GPC (18:1) | Lysophospholipid | HMDB0002815 | C03916 | 16081932 |
| 87 | 10-nonadecenoate (19:1n9) | Long Chain Monounsaturated Fatty Acid | HMDB0013622 | NA | 5312513 |
| 88 | 10-heptadecenoate (17:1n7) | Long Chain Monounsaturated Fatty Acid | HMDB0060038 | NA | 5312435 |
| 89 | eicosenoate (20:1) | Long Chain Monounsaturated Fatty Acid | HMDB0002231, HMDB0062436 | C16526 | 5.2828E+18 |
| 90 | linolenate [alpha or gamma; (18:3n3 or 6)] | Long Chain Polyunsaturated Fatty Acid (n3 and n6) | HMDB0003073, HMDB0001388 | C06426, C06427 | 5.2809E+13 |
| 91 | stearoylcarnitine (C18) | Fatty Acid Metabolism (Acyl Carnitine, Long Chain Saturated) | HMDB0000848 | NA | 3006797 |
| 92 | laurylcarnitine (C12) | Fatty Acid Metabolism (Acyl Carnitine, Medium Chain) | HMDB000225 | NA | 168381 |
| 93 | 1-linoleoyl-GPC (18:2) | Lysophospholipid | HMDB0010386 | C04100 | 11988421 |
| 94 | 1-arachidonylglycerol (20:4) | Monoacylglycerol | HMDB11578 | C13857 | 1.602E+14 |
| 95 | 1-linolenoylglycerol (18:3) | Monoacylglycerol | HMDB0011569 | NA | 53480978 |
| 96 | 3-hydroxydodecanedioate* | Fatty Acid, Dicarboxylate | HMDB0000413 | NA | 16663321 |
| 97 | 1-stearoyl-GPE (18:0) | Lysophospholipid | HMDB0011130 | NA | 9547068 |
| 98 | 1-stearoyl-GPG (18:0) | Lysophospholipid | NA | NA | NA |
| 99 | oleoylcarnitine (C18:1) | Fatty Acid Metabolism (Acyl Carnitine, Monounsaturated) | HMDB0005065 | NA | 6441392 |
| 100 | 1-palmitoleoyl-GPC (16:1)* | Lysophospholipid | HMDB0010383 | NA | 24779461 |
| 101 | 1-arachidonoyl-GPC (20:4n6)* | Lysophospholipid | HMDB0010395 | C05208 | 24779476 |
| 102 | 2-palmitoleoyl-GPC (16:1)* | Lysophospholipid | HMDB10383 | NA | 122164832 |
| 103 | 2-palmitoyl-GPC (16:0)* | Lysophospholipid | HMDB0061702 | NA | 15061532 |
| 104 | 1-palmitoyl-GPE (16:0) | Lysophospholipid | HMDB0011503 | NA | 9547069 |
| 105 | 1-oleoyl-GPE (18:1) | Lysophospholipid | HMDB0011506 | NA | 9547071 |
| 106 | 1-linoleoyl-GPE (18:2)* | Lysophospholipid | HMDB0011507 | NA | 52925130 |
| 107 | 1-arachidonoyl-GPE (20:4n6)* | Lysophospholipid | HMDB0011517 | NA | 42607465 |
| 108 | 2-hydroxypalmitate | Fatty Acid, Monohydroxy | HMDB0031057 | NA | 1.26E+20 |
| 109 | docosapentaenoate (n6 DPA; 22:5n6) | Long Chain Polyunsaturated Fatty Acid (n3 and n6) | HMDB0001976 | C16513 | 6441454 |
| 110 | tetradecanedioate (C14-DC) | Fatty Acid, Dicarboxylate | HMDB0000872 | NA | 13185 |
| 111 | hexadecanedioate (C16-DC) | Fatty Acid, Dicarboxylate | HMDB0000672 | C19615 | 10459 |
| 112 | octadecanedioate (C18-DC) | Fatty Acid, Dicarboxylate | HMDB0000782 | NA | 70095 |
| 113 | undecanedioate (C11-DC) | Fatty Acid, Dicarboxylate | HMDB0000888 | NA | 15816 |
| 114 | 1-myristoylglycerol (14:0) | Monoacylglycerol | HMDB0011561 | C01885 | 10957631 |
| 115 | glycerophosphoethanolamine | Phospholipid Metabolism | HMDB0000114 | C01233 | 123874 |
| 116 | 1-arachidonoyl-GPI (20:4)* | Lysophospholipid | HMDB0061690 | NA | 121596217 |
| 117 | 1-palmitoyl-GPI (16:0) | Lysophospholipid | HMDB0061695 | NA | 71296207 |
| 118 | deoxycarnitine | Carnitine Metabolism | HMDB0001161 | C01181 | 22620 |
| 119 | alpha-hydroxycaproate | Fatty Acid, Monohydroxy | HMDB0001624 | NA | 99824 |
| 120 | 3,4-dihydroxybutyrate | Fatty Acid, Dihydroxy | HMDB0000337 | NA | 150929 |
| 121 | hexanoylglutamine | Fatty Acid Metabolism (Acyl Glutamine) | NA | NA | NA |
| 122 | dihomo-linolenate (20:3n3 or n6) | Long Chain Polyunsaturated Fatty Acid (n3 and n6) | HMDB0002925 | C03242 | 5280581 |
| 123 | 1-oleoyl-GPI (18:1) | Lysophospholipid | HMDB0061693 | NA | 86289645 |
| 124 | 1-linoleoyl-GPI (18:2)* | Lysophospholipid | HMDB0240597 | NA | 11124828 |
| 125 | 1-stearoyl-2-oleoyl-GPE (18:0/18:1) | Phosphatidylethanolamine (PE) | HMDB0008993 | NA | 9546742 |
| 126 | 1-stearoyl-2-arachidonoyl-GPC (18:0/20:4) | Phosphatidylcholine (PC) | HMDB0008048 | NA | 16219824 |
| 127 | 1-palmitoyl-2-linoleoyl-GPE (16:0/18:2) | Phosphatidylethanolamine (PE) | HMDB0005322 | NA | 9546747 |
| 128 | sphinganine-1-phosphate | Sphingolipid Synthesis | HMDB0001383 | NA | 520 |
| 129 | glycosyl-N-stearoyl-sphingosine (d18:1/18:0) | Hexosylceramides (HCER) | NA | NA | NA |
| 130 | 2-hydroxyglutarate | Fatty Acid, Dicarboxylate | HMDB0059655 | C02630 | 43 |
| 131 | sphingomyelin (d18:1/18:1, d18:2/18:0) | Sphingomyelins | HMDB0012101 | NA | 6443882 |
| 132 | palmitoyl sphingomyelin (d18:1/16:0) | Sphingomyelins | HMDB0010169 | NA | 9939941 |
| 133 | 13-HODE + 9-HODE | Fatty Acid, Monohydroxy | HMDB0004670, HMDB0004667 | C14767, C14762 | 5.31E+31 |
| 134 | tridecenedioate (C13:1-DC)* | Fatty Acid, Dicarboxylate | NA | NA | NA |
| 135 | cis-4-decenoylcarnitine (C10:1) | Fatty Acid Metabolism (Acyl Carnitine, Monounsaturated) | HMDB0013205 | NA | 57357170 |
| 136 | 2S,3R-dihydroxybutyrate | Fatty Acid, Dihydroxy | HMDB0002453 | NA | 10964471 |
| 137 | (16 or 17)-methylstearate (a19:0 or i19:0) | Fatty Acid, Branched | HMDB0037397 | NA | 3083779 |
| 138 | 2R,3R-dihydroxybutyrate | Fatty Acid, Dihydroxy | HMDB0000498 | NA | 13120901 |
| 139 | cis-4-decenoate (10:1n6)* | Medium Chain Fatty Acid | HMDB0004980 | NA | 5282726 |
| 140 | 1-lignoceroyl-GPC (24:0) | Lysophospholipid | HMDB0010405 | NA | 24779481 |
| 141 | 1-(1-enyl-palmitoyl)-GPC (P-16:0)* | Lysoplasmalogen | HMDB0010407 | NA | 10917802 |
| 142 | (14 or 15)-methylpalmitate (a17:0 or i17:0) | Fatty Acid, Branched | HMDB0061859 | C16995 | 8.1812E+11 |
| 143 | eicosanedioate (C20-DC) | Fatty Acid, Dicarboxylate | NA | NA | 75502 |
| 144 | docosadioate (C22-DC) | Fatty Acid, Dicarboxylate | NA | C14240 | 7641 |
| 145 | 16-hydroxypalmitate | Fatty Acid, Monohydroxy | HMDB0006294 | C18218 | 10466 |
| 146 | oleoyl-linoleoyl-glycerol (18:1/18:2) [1] | Diacylglycerol | HMDB0007219 | NA | 14275396 |
| 147 | oleoyl-linoleoyl-glycerol (18:1/18:2) [2] | Diacylglycerol | HMDB0007219 | NA | 71433693 |
| 148 | 1-(1-enyl-palmitoyl)-GPE (P-16:0)* | Lysoplasmalogen | HMDB0011152 | NA | NA |
| 149 | 1-(1-enyl-stearoyl)-GPE (P-18:0)* | Lysoplasmalogen | HMDB0240598 | NA | NA |
| 150 | linoleoylcarnitine (C18:2)* | Fatty Acid Metabolism (Acyl Carnitine, Polyunsaturated) | HMDB0006469 | NA | 6450015 |
| 151 | trimethylamine N-oxide | Phospholipid Metabolism | HMDB0000925 | C01104 | 1145 |
| 152 | N-palmitoylglycine | Fatty Acid Metabolism (Acyl Glycine) | HMDB0013034 | NA | 151008 |
| 153 | 2-stearoyl-GPE (18:0)* | Lysophospholipid | HMDB0011129 | NA | 9.0658E+15 |
| 154 | (R)-3-hydroxybutyrylcarnitine | Fatty Acid Metabolism (Acyl Carnitine, Hydroxy) | HMDB0013127 | NA | 53481617 |
| 155 | margaroylcarnitine (C17)* | Fatty Acid Metabolism (Acyl Carnitine, Long Chain Saturated) | HMDB0006210 | NA | 53477803 |
| 156 | 2-hydroxydecanoate | Fatty Acid, Monohydroxy | HMDB0094656 | NA | 21488 |
| 157 | 2-aminooctanoate | Fatty Acid, Amino | HMDB0000991 | NA | 69522 |
| 158 | sphingomyelin (d18:1/14:0, d16:1/16:0)* | Sphingomyelins | HMDB0012097 | NA | 11433862 |
| 159 | sphingomyelin (d18:2/16:0, d18:1/16:1)* | Sphingomyelins | HMDB0240638, HMDB0240613 | NA | NA |
| 160 | 2-hydroxyphytanate* | Fatty Acid, Branched | HMDB0061666 | NA | 189026 |
| 161 | 2-aminoheptanoate | Fatty Acid, Amino | HMDB0094649 | NA | 227939 |
| 162 | 1-linolenoyl-GPC (18:3)* | Lysophospholipid | HMDB0010388 | C04100 | NA |
| 163 | 1-(1-enyl-oleoyl)-GPE (P-18:1)* | Lysoplasmalogen | NA | NA | NA |
| 164 | 1-oleoyl-GPG (18:1)* | Lysophospholipid | HMDB0240602 | NA | NA |
| 165 | 1-palmitoyl-GPG (16:0)* | Lysophospholipid | HMDB0240601 | NA | 3300276 |
| 166 | 9-hydroxystearate | Fatty Acid, Monohydroxy | HMDB0061661 | NA | 9570127 |
| 167 | sphingomyelin (d18:2/14:0, d18:1/14:1)* | Sphingomyelins | HMDB0240637, HMDB0240612 | NA | NA |
| 168 | sphingomyelin (d18:1/24:1, d18:2/24:0)* | Sphingomyelins | HMDB0012107 | NA | 44260126 |
| 169 | octadecenedioylcarnitine (C18:1-DC)* | Fatty Acid Metabolism (Acyl Carnitine, Dicarboxylate) | NA | NA | NA |
| 170 | octadecanedioylcarnitine (C18-DC)* | Fatty Acid Metabolism (Acyl Carnitine, Dicarboxylate) | NA | NA | NA |
| 171 | myristoleoylcarnitine (C14:1)* | Fatty Acid Metabolism (Acyl Carnitine, Monounsaturated) | HMDB0240588 | NA | 129691961 |
| 172 | 1-dihomo-linolenylglycerol (20:3) | Monoacylglycerol | HMDB0011577 | NA | NA |
| 173 | sphingomyelin (d18:1/20:0, d16:1/22:0)* | Sphingomyelins | HMDB0012102 | NA | 44260124 |
| 174 | sphingomyelin (d18:1/20:1, d18:2/20:0)* | Sphingomyelins | HMDB0240610, HMDB0240632 | NA | NA |
| 175 | sphingomyelin (d18:1/20:2, d18:2/20:1, d16:1/22:2)* | Sphingomyelins | NA | NA | NA |
| 176 | behenoyl sphingomyelin (d18:1/22:0)* | Sphingomyelins | HMDB0012103 | NA | 44260125 |
| 177 | sphingomyelin (d18:1/22:1, d18:2/22:0, d16:1/24:1)* | Sphingomyelins | HMDB0012104 | NA | NA |
| 178 | sphingomyelin (d18:1/22:2, d18:2/22:1, d16:1/24:2)* | Sphingomyelins | HMDB0240670, HMDB0240672, HMDB0240669 | NA | NA |
| 179 | lignoceroyl sphingomyelin (d18:1/24:0) | Sphingomyelins | HMDB0011697 | NA | NA |
| 180 | sphingomyelin (d17:1/16:0, d18:1/15:0, d16:1/17:0)* | Sphingomyelins | HMDB0240617, HMDB0240608 | NA | NA |
| 181 | 3-hydroxyhexanoate | Fatty Acid, Monohydroxy | HMDB0061652, HMDB0010718 | NA | 151492 |
| 182 | adipoylcarnitine (C6-DC) | Fatty Acid Metabolism (Acyl Carnitine, Dicarboxylate) | HMDB0061677 | NA | 71296139 |
| 183 | nonanoylcarnitine (C9) | Fatty Acid Metabolism (Acyl Carnitine, Medium Chain) | HMDB0013288 | NA | 91825642 |
| 184 | suberoylcarnitine (C8-DC) | Fatty Acid Metabolism (Acyl Carnitine, Dicarboxylate) | NA | NA | NA |
| 185 | 1,2-dilinoleoyl-GPC (18:2/18:2) | Phosphatidylcholine (PC) | HMDB0008138 | NA | 5288075 |
| 186 | 1-stearoyl-2-oleoyl-GPC (18:0/18:1) | Phosphatidylcholine (PC) | HMDB0008038 | NA | 24778825 |
| 187 | 1-palmitoyl-2-arachidonoyl-GPC (16:0/20:4n6) | Phosphatidylcholine (PC) | HMDB0007982 | C05208 | 10747814 |
| 188 | 1-palmitoyl-2-docosahexaenoyl-GPC (16:0/22:6) | Phosphatidylcholine (PC) | HMDB0007991 | NA | 6441886 |
| 189 | 1-stearoyl-2-docosahexaenoyl-GPC (18:0/22:6) | Phosphatidylcholine (PC) | HMDB0008057 | NA | 24778876 |
| 190 | 1-(1-enyl-stearoyl)-2-oleoyl-GPE (P-18:0/18:1) | Plasmalogen | HMDB0011375 | NA | 42607457 |
| 191 | sphingomyelin (d18:1/17:0, d17:1/18:0, d19:1/16:0) | Sphingomyelins | HMDB0240620, HMDB0240609, HMDB0240622 | NA | 46891763 |
| 192 | 1-palmitoyl-2-stearoyl-GPC (16:0/18:0) | Phosphatidylcholine (PC) | HMDB0007970 | C03889 | 24778686 |
| 193 | oleate/vaccenate (18:1) | Long Chain Monounsaturated Fatty Acid | HMDB0003231, HMDB0000573, HMDB0240219, HMDB0000207 | C21944, C08367, C01712, C00712 | 5.28E+25 |
| 194 | 1-palmitoleoylglycerol (16:1)* | Monoacylglycerol | HMDB0011565 | NA | NA |
| 195 | palmitoyl dihydrosphingomyelin (d18:0/16:0)* | Dihydrosphingomyelins | HMDB0010168 | NA | 9939965 |
| 196 | tricosanoyl sphingomyelin (d18:1/23:0)* | Sphingomyelins | HMDB0012105 | NA | NA |
| 197 | sphingomyelin (d18:2/23:0, d18:1/23:1, d17:1/24:1)* | Sphingomyelins | HMDB0240634, HMDB0011696, HMDB0240614 | NA | 134757653 |
| 198 | sphingomyelin (d18:2/24:1, d18:1/24:2)* | Sphingomyelins | HMDB0240636, HMDB0240615 | NA | 85336023 |
| 199 | 1-stearoyl-2-linoleoyl-GPE (18:0/18:2)* | Phosphatidylethanolamine (PE) | HMDB0008994 | NA | 9546749 |
| 200 | 1-stearoyl-2-arachidonoyl-GPE (18:0/20:4) | Phosphatidylethanolamine (PE) | HMDB0009003 | C05210 | 5289133 |
| 201 | 1-stearoyl-2-linoleoyl-GPC (18:0/18:2)* | Phosphatidylcholine (PC) | HMDB0008039 | NA | 6441487 |
| 202 | 1-palmitoyl-2-palmitoleoyl-GPC (16:0/16:1)* | Phosphatidylcholine (PC) | HMDB0007969 | NA | 6443788 |
| 203 | 1-palmitoyl-2-dihomo-linolenoyl-GPC (16:0/20:3n3 or 6)* | Phosphatidylcholine (PC) | NA | NA | NA |
| 204 | 1-palmitoyl-2-arachidonoyl-GPE (16:0/20:4)* | Phosphatidylethanolamine (PE) | HMDB0005323 | C05210 | 9546800 |
| 205 | 1-palmitoyl-2-docosahexaenoyl-GPE (16:0/22:6)* | Phosphatidylethanolamine (PE) | HMDB0008946 | NA | 9546799 |
| 206 | 1-stearoyl-2-docosahexaenoyl-GPE (18:0/22:6)* | Phosphatidylethanolamine (PE) | HMDB0009012 | NA | 9546798 |
| 207 | 1-palmitoyl-2-arachidonoyl-GPI (16:0/20:4)* | Phosphatidylinositol (PI) | HMDB0009789 | NA | NA |
| 208 | 1-stearoyl-2-linoleoyl-GPI (18:0/18:2) | Phosphatidylinositol (PI) | HMDB0009809 | NA | NA |
| 209 | 1-(1-enyl-stearoyl)-2-arachidonoyl-GPE (P-18:0/20:4)* | Plasmalogen | HMDB0005779 | NA | 9547058 |
| 210 | 1-(1-enyl-palmitoyl)-2-arachidonoyl-GPE (P-16:0/20:4)* | Plasmalogen | HMDB0011352 | NA | 52925126 |
| 211 | 1-(1-enyl-palmitoyl)-2-oleoyl-GPE (P-16:0/18:1)* | Plasmalogen | HMDB0011342 | NA | 52925128 |
| 212 | 1-(1-enyl-palmitoyl)-2-oleoyl-GPC (P-16:0/18:1)* | Plasmalogen | HMDB0007996 | NA | 34779384 |
| 213 | 1-(1-enyl-palmitoyl)-2-linoleoyl-GPC (P-16:0/18:2)* | Plasmalogen | HMDB0011211 | NA | 24779386 |
| 214 | 1-(1-enyl-palmitoyl)-2-arachidonoyl-GPC (P-16:0/20:4)* | Plasmalogen | HMDB0011220 | NA | NA |
| 215 | sphingomyelin (d18:1/21:0, d17:1/22:0, d16:1/23:0)* | Sphingomyelins | HMDB0240619, HMDB0240611, HMDB0240621 | NA | NA |
| 216 | behenoyl dihydrosphingomyelin (d18:0/22:0)* | Dihydrosphingomyelins | HMDB0012091 | NA | 44260132 |
| 217 | sphingomyelin (d18:0/18:0, d19:0/17:0)* | Dihydrosphingomyelins | HMDB0012087 | NA | 44260130 |
| 218 | N-palmitoyl-sphinganine (d18:0/16:0) | Dihydroceramides | HMDB11760 | NA | 5283572 |
| 219 | lactosyl-N-palmitoyl-sphingosine (d18:1/16:0) | Lactosylceramides (LCER) | HMDB0006750 | NA | 53477895 |
| 220 | myristoyl dihydrosphingomyelin (d18:0/14:0)* | Dihydrosphingomyelins | HMDB0012085 | NA | 44260138 |
| 221 | palmitoyl-linoleoyl-glycerol (16:0/18:2) [1]* | Diacylglycerol | HMDB0007103 | NA | 9543695 |
| 222 | palmitoyl-linoleoyl-glycerol (16:0/18:2) [2]* | Diacylglycerol | HMDB0007103 | NA | 131801744 |
| 223 | 1-palmitoyl-2-oleoyl-GPI (16:0/18:1)* | Phosphatidylinositol (PI) | HMDB0009783 | NA | 71296232 |
| 224 | 1-(1-enyl-palmitoyl)-2-linoleoyl-GPE (P-16:0/18:2)* | Plasmalogen | HMDB0011343 | NA | 52925127 |
| 225 | 1-oleoyl-2-linoleoyl-GPE (18:1/18:2)* | Phosphatidylethanolamine (PE) | HMDB0005349 | NA | 9546753 |
| 226 | 1-oleoyl-2-docosahexaenoyl-GPC (18:1/22:6)* | Phosphatidylcholine (PC) | HMDB0008123 | NA | 24778955 |
| 227 | 1-linoleoyl-2-arachidonoyl-GPC (18:2/20:4n6)* | Phosphatidylcholine (PC) | HMDB0008147 | C05208 | 24778979 |
| 228 | 1-myristoyl-2-arachidonoyl-GPC (14:0/20:4)* | Phosphatidylcholine (PC) | HMDB0007883 | NA | 24778634 |
| 229 | 1-(1-enyl-palmitoyl)-2-palmitoleoyl-GPC (P-16:0/16:1)* | Plasmalogen | HMDB0011207 | NA | 52923882 |
| 230 | 1-(1-enyl-palmitoyl)-2-palmitoyl-GPC (P-16:0/16:0)* | Plasmalogen | HMDB0011206 | NA | 11146967 |
| 231 | 1,2-dilinoleoyl-GPE (18:2/18:2)* | Phosphatidylethanolamine (PE) | HMDB0009093 | NA | 9546812 |
| 232 | 1-oleoyl-2-arachidonoyl-GPE (18:1/20:4)* | Phosphatidylethanolamine (PE) | HMDB0009069 | NA | 348281223 |
| 233 | 1-linoleoyl-2-arachidonoyl-GPE (18:2/20:4)* | Phosphatidylethanolamine (PE) | HMDB0009102 | NA | 348279332 |
| 234 | 1-(1-enyl-stearoyl)-2-linoleoyl-GPE (P-18:0/18:2)* | Plasmalogen | HMDB0011376 | NA | NA |
| 235 | 1-linoleoyl-GPG (18:2)* | Lysophospholipid | HMDB0240600 | NA | NA |
| 236 | palmitoylcholine | Fatty Acid Metabolism (Acyl Choline) | HMDB0240592 | NA | 151731 |
| 237 | (S)-3-hydroxybutyrylcarnitine | Fatty Acid Metabolism (Acyl Carnitine, Hydroxy) | HMDB0013127 | NA | 71464477 |
| 238 | glycosyl-N-palmitoyl-sphingosine (d18:1/16:0) | Hexosylceramides (HCER) | NA | NA | NA |
| 239 | arachidonoylcholine | Fatty Acid Metabolism (Acyl Choline) | HMDB0240583 | NA | 122198216 |
| 240 | 1-linoleoyl-2-linolenoyl-GPC (18:2/18:3)* | Phosphatidylcholine (PC) | HMDB0008141 | NA | 52922731 |
| 241 | hexadecadienoate (16:2n6) | Long Chain Polyunsaturated Fatty Acid (n3 and n6) | HMDB0000477 | NA | 13932172 |
| 242 | palmitoleoylcarnitine (C16:1)* | Fatty Acid Metabolism (Acyl Carnitine, Monounsaturated) | HMDB0013207 | NA | 71464547 |
| 243 | pimeloylcarnitine/3-methyladipoylcarnitine (C7-DC) | Fatty Acid Metabolism (Acyl Carnitine, Dicarboxylate) | NA | NA | NA |
| 244 | oleoyl-oleoyl-glycerol (18:1/18:1) [1]* | Diacylglycerol | HMDB0007218 | NA | 9543716 |
| 245 | oleoyl-oleoyl-glycerol (18:1/18:1) [2]* | Diacylglycerol | HMDB0007218 | NA | 9543716 |
| 246 | linoleoyl-arachidonoyl-glycerol (18:2/20:4) [1]* | Diacylglycerol | HMDB0007257 | NA | 9543796 |
| 247 | linoleoyl-arachidonoyl-glycerol (18:2/20:4) [2]* | Diacylglycerol | HMDB0007257 | NA | 9543796 |
| 248 | palmitoyl-arachidonoyl-glycerol (16:0/20:4) [1]* | Diacylglycerol | HMDB0007112 | NA | 9543736 |
| 249 | palmitoyl-arachidonoyl-glycerol (16:0/20:4) [2]* | Diacylglycerol | HMDB0007112 | NA | 9543736 |
| 250 | linoleoyl-linolenoyl-glycerol (18:2/18:3) [2]* | Diacylglycerol | HMDB0007250 | NA | 9543737 |
| 251 | oleoyl-arachidonoyl-glycerol (18:1/20:4) [1]* | Diacylglycerol | HMDB0007228 | NA | 9543786 |
| 252 | oleoyl-arachidonoyl-glycerol (18:1/20:4) [2]* | Diacylglycerol | HMDB0007228 | NA | 9543786 |
| 253 | linoleoyl-linoleoyl-glycerol (18:2/18:2) [1]* | Diacylglycerol | HMDB0007248 | NA | 9543729 |
| 254 | linoleoyl-linoleoyl-glycerol (18:2/18:2) [2]* | Diacylglycerol | HMDB0007248 | NA | 9543729 |
| 255 | N-palmitoyl-sphingadienine (d18:2/16:0)* | Ceramides | HMDB0240686 | NA | NA |
| 256 | lactosyl-N-nervonoyl-sphingosine (d18:1/24:1)* | Lactosylceramides (LCER) | HMDB0004872 | NA | NA |
| 257 | lactosyl-N-behenoyl-sphingosine (d18:1/22:0)* | Lactosylceramides (LCER) | HMDB0011594 | NA | 44260143 |
| 258 | glycosyl-N-behenoyl-sphingadienine (d18:2/22:0)* | Hexosylceramides (HCER) | NA | NA | NA |
| 259 | 2-hydroxybehenate | Fatty Acid, Monohydroxy | HMDB0061660 | NA | 193484 |
| 260 | 2-hydroxynervonate* | Fatty Acid, Monohydroxy | NA | NA | 5312783 |
| 261 | ceramide (d16:1/24:1, d18:1/22:1)* | Ceramides | NA | NA | NA |
| 262 | ceramide (d18:2/24:1, d18:1/24:2)* | Ceramides | HMDB0240679, HMDB0240680 | NA | NA |
| 263 | glycosyl ceramide (d18:2/24:1, d18:1/24:2)* | Hexosylceramides (HCER) | NA | NA | NA |
| 264 | glycosyl-N-tricosanoyl-sphingadienine (d18:2/23:0)* | Hexosylceramides (HCER) | NA | NA | NA |
| 265 | glycosyl-N-(2-hydroxynervonoyl)-sphingosine (d18:1/24:1(2OH))* | Hexosylceramides (HCER) | NA | NA | NA |
| 266 | linoleoylcholine* | Fatty Acid Metabolism (Acyl Choline) | HMDB0013213 | NA | 53481656 |
| 267 | sphingomyelin (d18:0/20:0, d16:0/22:0)* | Dihydrosphingomyelins | HMDB0012090 | NA | NA |
| 268 | sphingomyelin (d18:1/19:0, d19:1/18:0)* | Sphingomyelins | NA | NA | NA |
| 269 | sphingomyelin (d18:2/18:1)* | Sphingomyelins | HMDB0001348 | NA | 52931155 |
| 270 | sphingomyelin (d18:2/24:2)* | Sphingomyelins | HMDB0240644 | NA | NA |
| 271 | sphingomyelin (d18:2/21:0, d16:2/23:0)* | Sphingomyelins | HMDB0240676 | NA | NA |
| 272 | sphingomyelin (d18:2/23:1)* | Sphingomyelins | HMDB0240668 | NA | NA |
| 273 | sphingomyelin (d18:1/25:0, d19:0/24:1, d20:1/23:0, d19:1/24:0)* | Sphingomyelins | HMDB0240675, HMDB0240674, HMDB0240673, HMDB0240671 | NA | NA |
| 274 | sphingomyelin (d17:2/16:0, d18:2/15:0)* | Sphingomyelins | HMDB0240677 | NA | NA |
| 275 | linolenoylcarnitine (C18:3)* | Fatty Acid Metabolism (Acyl Carnitine, Polyunsaturated) | NA | NA | NA |
| 276 | lignoceroylcarnitine (C24)* | Fatty Acid Metabolism (Acyl Carnitine, Long Chain Saturated) | HMDB0240665 | NA | NA |
| 277 | cerotoylcarnitine (C26)* | Fatty Acid Metabolism (Acyl Carnitine, Long Chain Saturated) | HMDB0006347 | NA | 53477828 |
| 278 | ximenoylcarnitine (C26:1)* | Fatty Acid Metabolism (Acyl Carnitine, Monounsaturated) | NA | NA | NA |
| 279 | arachidonoylcarnitine (C20:4) | Fatty Acid Metabolism (Acyl Carnitine, Polyunsaturated) | HMDB0006455 | NA | 136212424 |
| 280 | eicosenoylcarnitine (C20:1)* | Fatty Acid Metabolism (Acyl Carnitine, Monounsaturated) | NA | NA | NA |
| 281 | dihomo-linoleoylcarnitine (C20:2)* | Fatty Acid Metabolism (Acyl Carnitine, Polyunsaturated) | NA | NA | NA |
| 282 | dihomo-linolenoylcarnitine (C20:3n3 or 6)* | Fatty Acid Metabolism (Acyl Carnitine, Polyunsaturated) | NA | NA | NA |
| 283 | glycosyl ceramide (d18:1/20:0, d16:1/22:0)* | Hexosylceramides (HCER) | NA | NA | NA |
| 284 | 5-dodecenoylcarnitine (C12:1) | Fatty Acid Metabolism (Acyl Carnitine, Monounsaturated) | HMDB13326 | NA | 91825571 |
| 285 | 2-butenoylglycine | Fatty Acid Metabolism (Acyl Glycine) | NA | NA | 6303498 |
| 286 | hydroxy-CMPF* | Fatty Acid, Dicarboxylate | NA | NA | NA |
| 287 | 3-hydroxyoleoylcarnitine | Fatty Acid Metabolism (Acyl Carnitine, Hydroxy) | NA | NA | 129637446 |
| 288 | trans-2-hexenoylglycine | Fatty Acid Metabolism (Acyl Glycine) | NA | NA | NA |
| 289 | 2-hydroxyarachidate* | Fatty Acid, Monohydroxy | NA | NA | 5225199 |
| 290 | 3-hydroxyoleate* | Fatty Acid, Monohydroxy | NA | NA | NA |
| 291 | dodecenedioate (C12:1-DC)* | Fatty Acid, Dicarboxylate | HMDB0000933 | NA | 5283028 |
| 292 | hexadecenedioate (C16:1-DC)* | Fatty Acid, Dicarboxylate | NA | NA | NA |
| 293 | octadecenedioate (C18:1-DC) | Fatty Acid, Dicarboxylate | NA | NA | 9543674 |
| 294 | heptenedioate (C7:1-DC)* | Fatty Acid, Dicarboxylate | NA | NA | NA |
| 295 | octadecadienedioate (C18:2-DC)* | Fatty Acid, Dicarboxylate | NA | NA | NA |
| 296 | 3-carboxy-4-methyl-5-pentyl-2-furanpropionate (3-CMPFP)** | Fatty Acid, Dicarboxylate | HMDB0061643 | NA | 194501 |
| 297 | N-acetyl-2-aminooctanoate* | Fatty Acid, Amino | HMDB0059745 | NA | 95555 |
| 298 | 3-hydroxybutyroylglycine** | Fatty Acid Metabolism (Acyl Glycine) | NA | NA | NA |
| 299 | sphingomyelin (d17:1/14:0, d16:1/15:0)* | Sphingomyelins | NA | NA | NA |
| 300 | tetradecadienoate (14:2)* | Long Chain Polyunsaturated Fatty Acid (n3 and n6) | HMDB0000560 | NA | 5312409 |
| 301 | 2-hydroxysebacate | Fatty Acid, Dicarboxylate | HMDB0000424 | NA | 128458 |
| 302 | dodecadienoate (12:2)* | Fatty Acid, Dicarboxylate | NA | NA | 25480 |
| 303 | 3-hydroxyhexanoylcarnitine (1) | Fatty Acid Metabolism (Acyl Carnitine, Hydroxy) | NA | NA | NA |
| 304 | hydroxypalmitoyl sphingomyelin (d18:1/16:0(OH))** | Sphingomyelins | NA | NA | NA |
| 305 | tetradecadienedioate (C14:2-DC)* | Fatty Acid, Dicarboxylate | NA | NA | 86018718 |
| 306 | eicosenedioate (C20:1-DC)* | Fatty Acid, Dicarboxylate | NA | NA | NA |
| 307 | undecenoylcarnitine (C11:1) | Fatty Acid Metabolism (Acyl Carnitine, Monounsaturated) | NA | NA | NA |
| 308 | 3-hydroxydecanoylcarnitine | Fatty Acid Metabolism (Acyl Carnitine, Hydroxy) | HMDB0061636 | NA | 121454166 |
| 309 | palmitoyl-sphingosine-phosphoethanolamine (d18:1/16:0) | Ceramide PEs | NA | NA | NA |
| 310 | picolinoylglycine | Fatty Acid Metabolism (Acyl Glycine) | HMDB0059766 | NA | 11788622 |
| 311 | branched chain 14:0 dicarboxylic acid** | Fatty Acid, Dicarboxylate | NA | NA | NA |
| 312 | (2 or 3)-decenoate (10:1n7 or n8) | Medium Chain Fatty Acid | NA | NA | NA |
| 313 | decadienedioic acid (C10:2-DC)** | Fatty Acid, Dicarboxylate | NA | NA | 17789717 |
| 314 | cis-3,4-methyleneheptanoate | Fatty Acid, Branched | NA | NA | NA |
| 315 | cis-3,4-methyleneheptanoylcarnitine | Fatty Acid Metabolism (Acyl Carnitine, Medium Chain) | NA | NA | NA |
| 316 | 3-hydroxyoctanoylcarnitine (1) | Fatty Acid Metabolism (Acyl Carnitine, Hydroxy) | NA | NA | NA |
| 317 | 3-hydroxyoctanoylcarnitine (2) | Fatty Acid Metabolism (Acyl Carnitine, Hydroxy) | NA | NA | NA |
| 318 | cis-3,4-methyleneheptanoylglycine | Fatty Acid Metabolism (Acyl Glycine) | NA | NA | NA |
