# Supplementary material for: Lysophospholipids Are Associated With Outcomes in Hospitalized Patients With Mild Traumatic Brain Injury
Source: J Neurotrauma. 2023 Dec 29;41(1-2):59–72. doi: 10.1089/neu.2023.0046 (PMC11071087; doi:10.1089/neu.2023.0046)
Supplement: Supplemental data [file Suppl_TableS4.docx]

Supplementary Table S4: Univariate models for lipid metabolites found to have significant effects on discharge GOSE.

|  | Chemical name | Univariate Model | FC (FDR P) | Up or Down Regulated |
| --- | --- | --- | --- | --- |
| **1** | **myo-inositol** | **-0.97±0.28 (0.021)** | **0.53 (0.021)** | **DOWN** |
| **2** | **methylmalonate (MMA)** | **-0.41±0.11 (0.010)** | **0.59 (0.010)** | **DOWN** |
| 3 | choline | -1.76±0.58 (0.039) | 0.80 (0.0031) | No |
| 4 | 1-palmitoyl-2-linoleoyl-GPC (16:0/18:2) | 0.095±0.028 (0.021) | 1.09 (0.021) | No |
| **5** | **hexanoylcarnitine (C6)** | **-1.64±0.56 (0.046)** | **0.47 (0.046)** | **DOWN** |
| **6** | **octanoylcarnitine (C8)** | **-1.81±0.57 (0.031)** | **0.38 (0.040)** | **DOWN** |
| 7 | 1-palmitoyl-GPC (16:0) | 0.12±0.029 (0.0083) | 1.28 (0.0083) | No |
| 8 | 1-stearoyl-GPC (18:0) | 0.10±0.031 (0.022) | 1.31 (0.022) | No |
| 9 | 1-oleoyl-GPC (18:1) | 0.17±0.056 (0.038) | 1.35 (0.038) | No |
| **10** | **1-linoleoyl-GPC (18:2)** | **0.29±0.068 (0.0083)** | **1.54 (0.0083)** | **UP** |
| 11 | 1-oleoyl-GPE (18:1) | 0.27±0.088 (0.039) | 1.496 (0.039) | No |
| **12** | **1-linoleoyl-GPE (18:2)** | **0.43±0.12 (0.021)** | **1.55 (0.021)** | **UP** |
| 13 | 1-arachidonoyl-GPE (20:4n6) | 0.28±0.095 (0.045) | 1.29 (0.045) | No |
| 14 | glycerophosphoethanolamine | 0.062±0.018 (0.021) | 1.31 (0.021) | No |
| **15** | **3,4-dihydroxybutyrate** | **-0.52±0.17 (0.045)** | **0.64 (0.021)** | **DOWN** |
| **16** | **cis-4-decenoylcarnitine (C10:1)** | **-0.71±0.16 (0.0051)** | **0.53 (0.0051)** | **DOWN** |
| 17 | 1-(1-enyl-palmitoyl)-GPC (P-16:0) | 0.090±0.029 (0.038) | 1.34 (0.038) | No |
| **18** | **1-linolenoyl-GPC (18:3)** | **0.38±0.10 (0.021)** | **1.89 (0.021)** | **UP** |
| **19** | **octadecanedioylcarnitine (C18-DC)** | **-1.58±0.52 (0.040)** | **0.50 (0.040)** | **DOWN** |
| **20** | **suberoylcarnitine (C8-DC)** | **-5.81±1.77 (0.027)** | **0.35 (0.027)** | **DOWN** |
| 21 | 1,2-dilinoleoyl-GPC (18:2/18:2) | 0.23±0.080 (0.047) | 1.37 (0.047) | No |
| 22 | 1-linoleoyl-2-arachidonoyl-GPC (18:2/20:4n6) | 0.20±0.064 (0.035) | 1.25 (0.035) | No |
| 23 | 1-oleoyl-2-arachidonoyl-GPE (18:1/20:4) | -0.54±0.19 (0.045) | 0.72 (0.045) | No |
| **24** | **(S)-3-hydroxybutyrylcarnitine** | **-2.19±0.62 (0.021)** | **0.48 (0.021)** | **DOWN** |
| **25** | **pimeloylcarnitine/3-methyladipoylcarnitine (C7-DC)** | **-1.72±0.53 (0.030)** | **0.45 (0.030)** | **DOWN** |
| 26 | sphingomyelin (d18:2/21:0, d16:2/23:0) | -0.34±0.12 (0.045) | 0.79 (0.045) | NO |
| **27** | **3-hydroxyhexanoylcarnitine (1)** | **-1.95±0.50 (0.010)** | **0.48 (0.010)** | **DOWN** |
| 28 | undecenoylcarnitine (C11:1) | -0.61±0.17 (0.021) | 0.67 (0.021) | No |
| **29** | **3-hydroxydecanoylcarnitine** | **-0.68±0.20 (0.021)** | **0.62 (0.021)** | **DOWN** |
| **30** | **cis-3,4-methyleneheptanoylcarnitine** | **-0.72±0.18 (0.010)** | **0.60 (0.010)** | **DOWN** |
| **31** | **3-hydroxyoctanoylcarnitine (1)** | **-0.71±0.21 (0.022)** | **0.61 (0.022)** | **DOWN** |
