# Supplementary material for: Lysophospholipids Are Associated With Outcomes in Hospitalized Patients With Mild Traumatic Brain Injury
Source: J Neurotrauma. 2023 Dec 29;41(1-2):59–72. doi: 10.1089/neu.2023.0046 (PMC11071087; doi:10.1089/neu.2023.0046)
Supplement: Supplemental data [file Suppl_TableS5.docx]

Supplementary Table S5: Levels of three key lysophospholipids among mTBI patients with or without type 2 diabetes or hyperlipidemia.

| **Lyosphospholipid** | **Type 2 Diabetes** | | ***P*-Value** | **Hyperlipidemia** | | ***P*-Value** |
| --- | --- | --- | --- | --- | --- | --- |
|  | **No** | **Yes** |  | **No** | **Yes** |  |
| 1-linoleoyl-GPC (18:2) | 0.731±0.369* | 0.642±0.290 | 0.279 | 0.714±0.351 | 0.683±0.320 | 0.683 |
| 1-linoleoyl-GPE (18:2) | 1.10±0.698 | 0.938±0.463 | 0.257 | 1.03±0.609 | 1.03±0.586 | 0.989 |
| 1-linolenoyl-GPC (18:3) | 0.695±0.515 | 0.503±0.248 | 0.128 | 0.679±0.583 | 0.567±0.339 | 0.270 |

*Values are means ± standard deviations.
