# Supplementary material for: Lysophospholipids Are Associated With Outcomes in Hospitalized Patients With Mild Traumatic Brain Injury
Source: J Neurotrauma. 2023 Dec 29;41(1-2):59–72. doi: 10.1089/neu.2023.0046 (PMC11071087; doi:10.1089/neu.2023.0046)
Supplement: Supplemental data [file Suppl_FigureS1.docx]

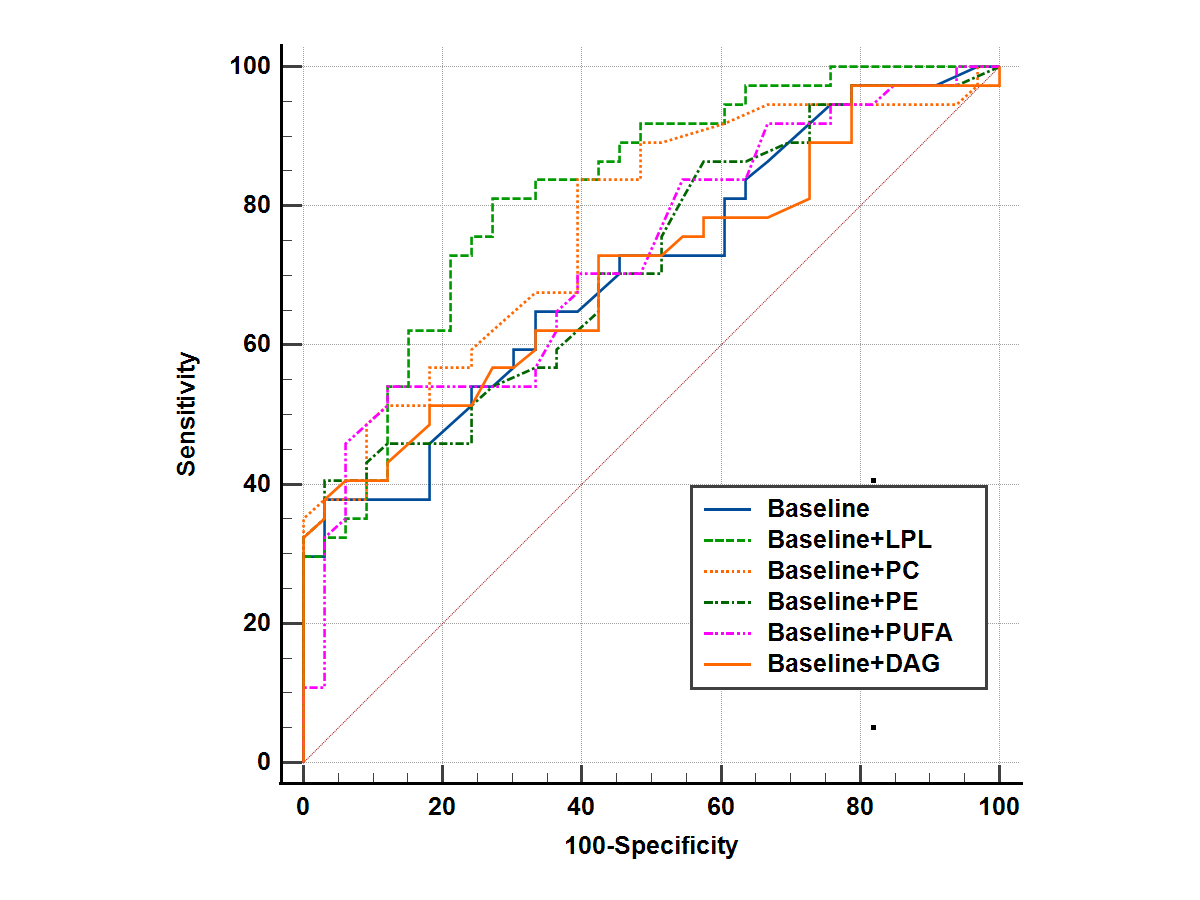
Supplementary Figure S1. ROC curves for 6-month GOSE prediction. Baseline model includes age and sex. Additional models add principal components consisting of metabolites from each pathway (LPL, PC, PE, PUFA, DAG) to the baseline model. Only the baseline model with the addition of LPL resulted in a significant improvement over the baseline model. Abbreviations: lysophospholipids (LPL), phosphatidylcholine (PC), phosphatidylethanolamine (PE), polyunsaturated fatty acids (PUFA), diacylglycerol (DAG).
