# Supplementary material for: Lysophospholipids Are Associated With Outcomes in Hospitalized Patients With Mild Traumatic Brain Injury
Source: J Neurotrauma. 2023 Dec 29;41(1-2):59–72. doi: 10.1089/neu.2023.0046 (PMC11071087; doi:10.1089/neu.2023.0046)
Supplement: Supplemental data [file Suppl_TableS6.docx]

Supplementary Table S6: Correlations between choline and lysophospholipids, phosphatidylcholines, phosphatidylserine, and metabolites in phospholipid metabolism. Metabolites in bold were also found to have associations with outcomes.

|  | **Control** | | **Early** | | **Late** | |
| --- | --- | --- | --- | --- | --- | --- |
| **Metabolite** | **ρ*** | ***P*** | **ρ** | ***P*** | **ρ** | ***P*** |
| 1-stearoyl-GPI (18:0) | 0.055 | 0.742 | -0.012 | 0.916 | -0.316 | 0.078 |
| 1-palmitoyl-GPC (16:0) | 0.410 | 0.010 | 0.001 | 0.990 | -0.315 | 0.079 |
| 1-stearoyl-GPC (18:0) | 0.348 | 0.030 | 0.014 | 0.900 | -0.233 | 0.200 |
| 1-oleoyl-GPC (18:1) | 0.301 | 0.062 | 0.036 | 0.748 | -0.270 | 0.134 |
| **1-linoleoyl-GPC (18:2)** | **0.280** | **0.084** | **-0.020** | **0.860** | **-0.255** | **0.159** |
| 1-stearoyl-GPE (18:0) | 0.249 | 0.126 | 0.017 | 0.881 | -0.169 | 0.355 |
| 1-stearoyl-GPG (18:0) | 0.305 | 0.059 | -0.005 | 0.967 | -0.119 | 0.515 |
| 1-palmitoleoyl-GPC* (16:1) | 0.269 | 0.098 | -0.071 | 0.521 | -0.433 | 0.013 |
| 1-arachidonoyl-GPC* (20:4) | 0.439 | 0.005 | 0.172 | 0.118 | 0.099 | 0.591 |
| 2-palmitoleoyl-GPC* (16:1) | 0.212 | 0.194 | 0.004 | 0.975 | -0.232 | 0.202 |
| 2-palmitoyl-GPC* (16:0) | 0.170 | 0.302 | -0.104 | 0.348 | -0.025 | 0.893 |
| 1-palmitoyl-GPE (16:0) | 0.066 | 0.688 | 0.054 | 0.624 | -0.195 | 0.284 |
| 1-oleoyl-GPE (18:1) | 0.262 | 0.108 | 0.074 | 0.505 | -0.087 | 0.637 |
| **1-linoleoyl-GPE (18:2)** | **0.258** | **0.113** | **-0.009** | **0.934** | **-0.245** | **0.176** |
| 1-arachidonoyl-GPE (20:4n6 | 0.462 | 0.003 | 0.095 | 0.388 | -0.002 | 0.993 |
| 1-arachidonoyl-GPI* (20:4) | 0.260 | 0.109 | 0.084 | 0.449 | 0.015 | 0.937 |
| 1-palmitoyl-GPI* (16:0) | -0.045 | 0.785 | -0.029 | 0.797 | -0.547 | 0.001 |
| 1-oleoyl-GPI (18:1) | -0.043 | 0.795 | -0.104 | 0.346 | -0.371 | 0.036 |
| 1-linoleoyl-GPI* (18:2) | -0.082 | 0.618 | -0.100 | 0.366 | -0.116 | 0.526 |
| 1-lignoceroyl-GPC (24:0) | 0.049 | 0.766 | 0.173 | 0.116 | -0.177 | 0.332 |
| 2-stearoyl-GPE (18:0) | 0.044 | 0.790 | -0.118 | 0.285 | -0.008 | 0.965 |
| **1-linolenoyl-GPC (18:3)** | **0.279** | **0.085** | **-0.130** | **0.239** | **-0.391** | **0.027** |
| 1-oleoyl-GPG (18:1) | 0.126 | 0.444 | 0.157 | 0.154 | 0.005 | 0.979 |
| 1-palmitoyl-GPG (16:0) | 0.116 | 0.481 | 0.127 | 0.250 | -0.090 | 0.624 |
| 1-linoleoyl-GPG (18:2) | 0.147 | 0.371 | 0.130 | 0.237 | 0.150 | 0.412 |
| 1-stearoyl-2-oleoyl-GPS (18:0/18:1) | 0.304 | 0.060 | 0.140 | 0.206 | 0.180 | 0.324 |
| 1-palmitoyl-2-linoleoyl-GPC (16:0/18:2) | 0.039 | 0.816 | -0.160 | 0.145 | -0.241 | 0.184 |
| 1-palmitoyl-2-oleoyl-GPC (16:0/18:1) | 0.083 | 0.614 | -0.025 | 0.820 | -0.273 | 0.131 |
| 1,2-dipalmitoyl-GPC (16:0/16:0) | -0.003 | 0.985 | -0.002 | 0.987 | -0.046 | 0.805 |
| 1-myristoyl-2-palmitoyl-GPC (14:0/16:0) | -0.092 | 0.578 | -0.244 | 0.025 | -0.371 | 0.036 |
| 1-stearoyl-2-arachidonoyl-GPC (18:0/20:4) | 0.315 | 0.051 | 0.293 | 0.007 | 0.341 | 0.056 |
| 1,2-dilinoleoyl-GPC (18:2/18:2) | -0.132 | 0.424 | -0.191 | 0.082 | -0.374 | 0.035 |
| 1-stearoyl-2-oleoyl-GPC (18:0/18:1) | 0.176 | 0.284 | 0.011 | 0.919 | -0.295 | 0.101 |
| 1-palmitoyl-2-arachidonoyl-GPC (16:0/20:4n6) | 0.273 | 0.092 | 0.225 | 0.040 | 0.134 | 0.466 |
| 1-palmitoyl-2-docosahexaenoyl-GPC (16:0/22:6) | 0.066 | 0.690 | 0.207 | 0.059 | 0.012 | 0.948 |
| 1-stearoyl-2-docosahexaenoyl-GPC (18:0/22:6) | 0.056 | 0.733 | 0.299 | 0.006 | 0.136 | 0.459 |
| 1-palmitoyl-2-stearoyl-GPC (16:0/18:0) | 0.198 | 0.228 | 0.038 | 0.733 | -0.176 | 0.334 |
| 1-stearoyl-2-linoleoyl-GPC (18:0/18:2) | 0.090 | 0.584 | -0.051 | 0.642 | -0.125 | 0.494 |
| 1-palmitoyl-2-palmitoleoyl-GPC (16:0/16:1) | -0.055 | 0.739 | -0.188 | 0.087 | -0.403 | 0.022 |
| 1-palmitoyl-2-dihomo-linolenoyl-GPC (16:0/20:3n3) | 0.136 | 0.408 | -0.005 | 0.966 | -0.220 | 0.227 |
| 1-oleoyl-2-docosahexaenoyl-GPC (18:1/22:6) | -0.067 | 0.683 | 0.423 | 0.000 | 0.165 | 0.368 |
| 1-linoleoyl-2-arachidonoyl-GPC (18:2/20:4n6) | 0.170 | 0.300 | -0.081 | 0.466 | -0.200 | 0.272 |
| 1-myristoyl-2-arachidonoyl-GPC (14:0/20:4) | 0.087 | 0.599 | -0.126 | 0.252 | -0.391 | 0.027 |
| 1-linoleoyl-2-linolenoyl-GPC (18:2/18:3) | 0.001 | 0.994 | -0.171 | 0.023 | -0.336 | 0.060 |
| 1-palmitoleoyl-2-linolenoyl-GPC (16:1/18:3) | -0.213 | 0.193 | -0.137 | 0.213 | -0.155 | 0.397 |
| phosphocholine | 0.053 | 0.748 | -0.070 | 0.529 | -0.239 | 0.188 |
| phosphoethanolamine (PE) | 0.071 | 0.669 | 0.230 | 0.035 | 0.088 | 0.633 |
| glycerophosphorylcholine (GPC) | 0.355 | 0.027 | -0.140 | 0.089 | -0.294 | 0.103 |
| glycerophosphoethanolamine | 0.236 | 0.149 | -0.179 | 0.104 | -0.240 | 0.185 |

*Pearson correlation coefficients
