# Supplementary material for: Lysophospholipids Are Associated With Outcomes in Hospitalized Patients With Mild Traumatic Brain Injury
Source: J Neurotrauma. 2023 Dec 29;41(1-2):59–72. doi: 10.1089/neu.2023.0046 (PMC11071087; doi:10.1089/neu.2023.0046)
Supplement: Supplemental data [file Suppl_FigureS2.docx]

**
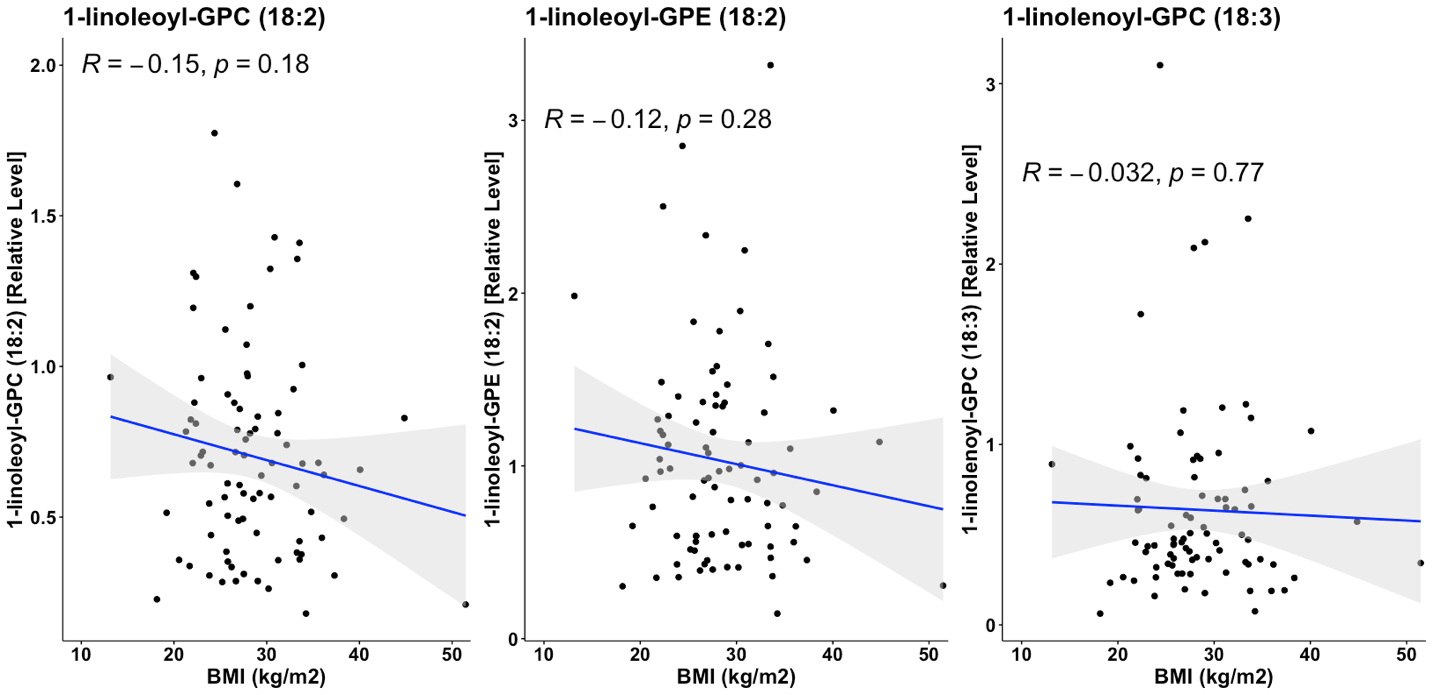
**

Supplementary Figure S2: Figure demonstrating the relationships between levels of three key lysophospholipids and body mass index (BMI) among mild TBI subjects.
