# Supplementary material for: Lysophospholipids Are Associated With Outcomes in Hospitalized Patients With Mild Traumatic Brain Injury
Source: J Neurotrauma. 2023 Dec 29;41(1-2):59–72. doi: 10.1089/neu.2023.0046 (PMC11071087; doi:10.1089/neu.2023.0046)
Supplement: Supplemental data [file Suppl_TableS7.docx]

Supplementary Table 7: List of metabolites included in each pathway for PCA analysis.

| LPL | PC | PE | DAG | PUFA |
| --- | --- | --- | --- | --- |
| 1-stearoyl-GPI (18:0) | 1-palmitoyl-2-linoleoyl-GPC (16:0/18:2) | 1-palmitoyl-2-oleoyl-GPE (16:0/18:1) | oleoyl-linoleoyl-glycerol (18:1/18:2) [1] | linoleate (18:2n6) |
| 1-palmitoyl-GPC (16:0) | 1-palmitoyl-2-oleoyl-GPC (16:0/18:1) | 1-stearoyl-2-oleoyl-GPE (18:0/18:1) | oleoyl-linoleoyl-glycerol (18:1/18:2) [2] | arachidonate (20:4n6) |
| 1-stearoyl-GPC (18:0) | 1,2-dipalmitoyl-GPC (16:0/16:0) | 1-palmitoyl-2-linoleoyl-GPE (16:0/18:2) | palmitoyl-linoleoyl-glycerol (16:0/18:2) [1]* | dihomolinoleate (20:2n6) |
| 1-oleoyl-GPC (18:1) | 1-myristoyl-2-palmitoyl-GPC (14:0/16:0) | 1-stearoyl-2-linoleoyl-GPE (18:0/18:2)* | palmitoyl-linoleoyl-glycerol (16:0/18:2) [2]* | eicosapentaenoate (EPA; 20:5n3) |
| 1-linoleoyl-GPC (18:2) | 1-stearoyl-2-arachidonoyl-GPC (18:0/20:4) | 1-stearoyl-2-arachidonoyl-GPE (18:0/20:4) | oleoyl-oleoyl-glycerol (18:1/18:1) [1]* | docosahexaenoate (DHA; 22:6n3) |
| 1-stearoyl-GPE (18:0) | 1,2-dilinoleoyl-GPC (18:2/18:2) | 1-palmitoyl-2-arachidonoyl-GPE (16:0/20:4)* | oleoyl-oleoyl-glycerol (18:1/18:1) [2]* | docosapentaenoate (DPA; 22:5n3) |
| 1-stearoyl-GPG (18:0) | 1-stearoyl-2-oleoyl-GPC (18:0/18:1) | 1-palmitoyl-2-docosahexaenoyl-GPE (16:0/22:6)* | linoleoyl-arachidonoyl-glycerol (18:2/20:4) [1]* | docosadienoate (22:2n6) |
| 1-palmitoleoyl-GPC* (16:1)* | 1-palmitoyl-2-arachidonoyl-GPC (16:0/20:4n6) | 1-stearoyl-2-docosahexaenoyl-GPE (18:0/22:6)* | linoleoyl-arachidonoyl-glycerol (18:2/20:4) [2]* | adrenate (22:4n6) |
| 1-arachidonoyl-GPC* (20:4)* | 1-palmitoyl-2-docosahexaenoyl-GPC (16:0/22:6) | 1-oleoyl-2-linoleoyl-GPE (18:1/18:2)* | palmitoyl-arachidonoyl-glycerol (16:0/20:4) [1]* | stearidonate (18:4n3) |
| 2-palmitoleoyl-GPC* (16:1)* | 1-stearoyl-2-docosahexaenoyl-GPC (18:0/22:6) | 1,2-dilinoleoyl-GPE (18:2/18:2)* | palmitoyl-arachidonoyl-glycerol (16:0/20:4) [2]* | linolenate (18:3n3 or 3n6) |
| 2-palmitoyl-GPC* (16:0)* | 1-palmitoyl-2-stearoyl-GPC (16:0/18:0) | 1-oleoyl-2-arachidonoyl-GPE (18:1/20:4)* | linoleoyl-linolenoyl-glycerol (18:2/18:3) [2]* | docosapentaenoate (n6 DPA; 22:5n6) |
| 1-palmitoyl-GPE (16:0) | 1-stearoyl-2-linoleoyl-GPC (18:0/18:2)* | 1-linoleoyl-2-arachidonoyl-GPE (18:2/20:4)* | oleoyl-arachidonoyl-glycerol (18:1/20:4) [1]* | dihomolinolenate (20:3n3 or 3n6) |
| 1-oleoyl-GPE (18:1) | 1-palmitoyl-2-palmitoleoyl-GPC (16:0/16:1)* |  | oleoyl-arachidonoyl-glycerol (18:1/20:4) [2]* | hexadecadienoate (16:2n6) |
| 1-linoleoyl-GPE (18:2)* | 1-palmitoyl-2-dihomo-linolenoyl-GPC (16:0/20:3n3 or 6)* |  | linoleoyl-linoleoyl-glycerol (18:2/18:2) [1]* | tetradecadienoate (14:2)* |
| 1-arachidonoyl-GPE (20:4n6)* | 1-oleoyl-2-docosahexaenoyl-GPC (18:1/22:6)* |  | linoleoyl-linoleoyl-glycerol (18:2/18:2) [2]* |  |
| 1-arachidonoyl-GPI* (20:4)* | 1-linoleoyl-2-arachidonoyl-GPC (18:2/20:4n6)* |  |  |  |
| 1-palmitoyl-GPI* (16:0) | 1-myristoyl-2-arachidonoyl-GPC (14:0/20:4)* |  |  |  |
| 1-oleoyl-GPI (18:1) | 1-linoleoyl-2-linolenoyl-GPC (18:2/18:3)* |  |  |  |
| 1-linoleoyl-GPI* (18:2)* |  |  |  |  |
| 1-lignoceroyl-GPC (24:0) |  |  |  |  |
| 2-stearoyl-GPE (18:0)* |  |  |  |  |
| 1-linolenoyl-GPC (18:3)* |  |  |  |  |
| 1-oleoyl-GPG (18:1)* |  |  |  |  |
| 1-palmitoyl-GPG (16:0)* |  |  |  |  |
| 1-linoleoyl-GPG (18:2)* |  |  |  |  |

Abbreviations: lysophospholipids (LPL), phosphatidylcholine (PC), phosphatidylethanolamine (PE), diacyclglycerol (DAG), polyunsaturated fatty acids (PUFA).
